# Extensive Local Gene Duplication and Functional Divergence among Paralogs in Atlantic Salmon

**DOI:** 10.1093/gbe/evu131

**Published:** 2014-06-19

**Authors:** Ian A. Warren, Kate L. Ciborowski, Elisa Casadei, David G. Hazlerigg, Sam Martin, William C. Jordan, Seirian Sumner

**Affiliations:** ^1^School of Biological Sciences, University of Bristol, United Kingdom; ^2^Institute of Zoology, Zoological Society of London, London, United Kingdom; ^3^Institute of Biological and Environmental Sciences, University of Aberdeen, United Kingdom; ^4^Department of Arctic and Marine Biology, Faculty of BioSciences Fisheries & Economy, University of Tromsø, Norway

**Keywords:** Atlantic salmon, gene duplication, whole-genome duplication, genome evolution, transcriptome

## Abstract

Many organisms can generate alternative phenotypes from the same genome, enabling individuals to exploit diverse and variable environments. A prevailing hypothesis is that such adaptation has been favored by gene duplication events, which generate redundant genomic material that may evolve divergent functions. Vertebrate examples of recent whole-genome duplications are sparse although one example is the salmonids, which have undergone a whole-genome duplication event within the last 100 Myr. The life-cycle of the Atlantic salmon, *Salmo salar*, depends on the ability to produce alternating phenotypes from the same genome, to facilitate migration and maintain its anadromous life history. Here, we investigate the hypothesis that genome-wide and local gene duplication events have contributed to the salmonid adaptation. We used high-throughput sequencing to characterize the transcriptomes of three key organs involved in regulating migration in *S. salar*: Brain, pituitary, and olfactory epithelium. We identified over 10,000 undescribed *S. salar* sequences and designed an analytic workflow to distinguish between paralogs originating from local gene duplication events or from whole-genome duplication events. These data reveal that substantial local gene duplications took place shortly after the whole-genome duplication event. Many of the identified paralog pairs have either diverged in function or become noncoding. Future functional genomics studies will reveal to what extent this rich source of divergence in genetic sequence is likely to have facilitated the evolution of extreme phenotypic plasticity required for an anadromous life-cycle.

## Introduction

Over the course of evolution, organisms have adapted to an astonishing range of environments and have implemented a plethora of life-history strategies in order to do so. Such examples range from Arctic foxes (*Alopex lagopus*), which change fur color with the seasons (Hersteinsson 1989), to the behavioral and morphological plasticity associated with alternative phenotypes observed in social insects (Ferreira et al. 2013). Understanding how such variation arises at the level of the genes is critical for understanding the process of adaptation. What changes are required to adapt to an environment, sometimes incorporating multiple ecotypes in a single generation?

The Atlantic salmon, *Salmo salar*, boasts a fascinating life history, involving a dramatic phenotypic switch in response to internal and environmental cues. *Salmo salar* spawn in freshwater streams, and the young spend 1–4 years in the natal stream, before migrating out to sea (Folmar and Dickhoff 1980). Migration requires metamorphosis from the freshwater morph (parr) to the saltwater morph (smolt). The parr–smolt transformation (PST) and subsequent migration occurs in spring, and its onset is dependent on the metabolic and physiological condition of the individual, as well as photoperiod cues (Folmar and Dickhoff 1980). Adult fish remain at sea feeding for 1–4 years. Mature salmon return with remarkable fidelity to their natal stream to reproduce, a behavior which relies heavily on olfaction (Hasler et al. 1978). The molecular mechanisms underlying PST and migration are poorly understood although it is likely to involve changes in gene expression and gene function in regions of the body involved in processing environmental and developmental cues, such as the hypothalamus, pituitary and olfactory epithelium (Prunet et al. 1989; Nevitt and Dittman 1998; Falcón et al. 2007; Nordgarden et al. 2007). To date, we lack information on the genes expressed in these tissues in *S. salar* (Rise et al. 2004).

Genome adaptation to a new environment and life-history strategy, such as anadromy in salmon where multiple ecotypes are required, involves one or both of two processes: 1) Rewiring of ancestral regulatory networks that subsequently results in altered gene expression; 2) changes in the proteins coded by the genes themselves, including the acquisition of new genes, and transitions to nonprotein-coding functions (Stern and Orgogozo 2008). The vast majority of genes in a genome are under strong purifying selection, thus preventing changes in both regulatory and coding sequences. One mechanism by which such purifying selection can be relaxed is through gene duplication (Hurles 2004; Sémon and Wolfe 2007; Peer et al. 2009). A change in gene regulation, and/or coding sequence would then be plausible for a redundant copy. Gene duplication events, both of whole genomes and of individual (local) genes, are expected to be one of the most important mechanisms for generating phenotypic diversity (Kondrashov et al. 2002; Kellogg 2003; Zhang 2003; Emes and Yang 2008; Santini et al. 2009) and in some instances discrete phenotypic plasticity (Lynch and Conery 2000; Chen et al. 2008).

Whether paralogs diverge in function and how this may happen appears to depend on many factors. There are three main theories concerning the fate of paralogs: 1) The dosage balance model, where an appropriate level of gene product is maintained by both paralogs, therefore they do not diverge greatly in function or expression pattern (Birchler and Veitia 2007; Hughes et al. 2007; Birchler and Veitia 2011); 2) subfunctionalization, where the roles of the ancestral gene are subdivided between the duplicates, resulting in (and caused by) changes in both sequence and expression pattern; 3) neofunctionalization, where one or both of the duplicates gain functions not present in the ancestral gene. There is evidence for all three processes (Papp et al. 2003; Guo et al. 2013; Plata and Vitkup 2014) but the relative prevalence of each process is affected by circumstance, such as the source of duplications, genomic architecture, the function of sequence that has been duplicated, and selection pressures (Lu et al. 2012; Zhu et al. 2012; Berthelot et al. 2014). Therefore to understand how paralogs will evolve requires understanding of the genomic architecture, how the duplications occurred and the types of sequences involved. Most studies on the effects of genome duplications have focused on yeast and plants; far less is known about what happens in vertebrate systems due to a paucity of study systems.

*Salmo salar*, and other species in the salmonid lineage, provide an excellent model system for studying the role of gene duplication(s) and adaptation, as the common salmonid ancestor underwent a whole-genome duplication (WGD) event between 50 and 100 Ma (Ohno 1970; Allendorf and Thorgaard 1984; Alexandrou et al. 2013; Berthelot et al. 2014; Macqueen and Johnston 2014). A high paralog retention rate in salmonids (25–75%) (Bailey et al. 1978) suggests that duplicated genes play important roles in their biology. A recent study has also suggested that more recent local gene duplication (LGD) events may be prevalent within the *S. salar* genome (Koop and Davidson 2008) due to the high nucleotide similarity observed between potential paralog pairs. However, this theory requires further testing. Understanding the different types of duplicates present and how they have evolved in an individual species is key to understanding how they contribute to the species’ adaptation. However, difficulties arise in such analyses when trying to distinguish between the origins of a duplication as well as between true duplicate genes, isoforms and allelic variants, which can lead to inaccurate estimations of the types and effects of duplication events.

Despite the economic and ecological importance of *S. salar*, current genomic resources for this species are surprisingly incomplete. A scaffold genome (Davidson et al. 2010), several EST (expressed sequence tag) libraries and microarray chips (Rise et al. 2004; Martin et al. 2007; Koop et al. 2008; Micallef et al. 2012), microsatellites (Ng et al. 2005; Vasemägi et al. 2005), single nucleotide polymorphisms, and linkage maps (Moen et al. 2008; Lien et al. 2011) are available. Yet we lack comprehensive information on transcription across all tissues. In particular, understanding how transcription regulates and coordinates the physiological and behavioral changes of *S. salar*’s complex life history is of vital cultural and economic importance, for example in advising management of fish stocks, breeding conditions, and predicting how environmental change may affect *S. salar* populations in the future.

In this study, using RNA from juvenile premigratory Atlantic salmon (parr) we first generated tissue-specific transcriptomes for three tissues important for migration: The brain (including the hypothalamus), the pituitary gland, and the olfactory epithelium. We went on to use our data set to demonstrate that paralog genes derived from both WGD and LGD events are present, and that a degree of functional divergence is occurring between sequences of a paralog pair. Our results contribute to understanding the nature of the duplication events that have occurred in the ancestry of *S. salar*, and assessing their potential contribution to phenotypic plasticity associated with an anadromous life history.

## Materials and Methods

### Fish Rearing and Sample Collection

All fish used in this study were sired from wild crosses of salmon from the River Tay, Scotland. Crosses were generated using ten female and ten male fish to remove any family bias. Fish were maintained at the Marine Scotland Science Freshwater facility Almond bank, Scotland. Samples were taken from fish during two stages in parr development (early and late). Tissue was collected from five individuals for each tissue (brain, olfactory epithelium, and pituitary gland) at each stage (early and late). Fish were sacrificed by schedule 1 killing using overdose in anesthetic (benzocane).

The entire brain, olfactory epithelium, and pituitary gland were extracted from each fish, and each separate tissue was placed immediately into 1 ml of RNALater (Applied Biosystems, Warrington, UK) and stored at 4 °C for 24 h before being transferred to −80 °C until RNA extraction.

### RNA Extraction, cDNA Library Preparation, and Sequencing

We extracted total RNA from each tissue using TRI reagent (Applied Biosystems, Warrington, UK) using the manufacturer’s recommendations. Extracted RNA was quantified using an ND-1000 Nanodrop spectrophotometer (Labtech Int., East Sussex, UK). An Agilent 2100 Bioanalyzer (Agilent Technologies, Cheshire, UK) was used to assess RNA integrity and purity.

For each tissue (brain, olfactory epithelium, and pituitary gland), a pool of 5 μg total RNA composed of an equimolar mixture of RNA from each extraction was used for cDNA library preparation. The cDNA libraries were synthesized using Evrogen SMART technology cDNA synthesis service (Evrogen, Moscow, Russia). High levels of expression of a few genes are expected in the pituitary and so this tissue was normalized before sequencing. Sequencing of the cDNA libraries was carried out on a GS FLX 454 sequencer (Roche, Switzerland) with one full plate being used for each tissue library. Synthesis of cDNA libraries and 454-sequencing was carried out by The GenePool (University of Edinburgh, UK). Raw reads were deposited to the National Center for Biotechnology Information (NCBI) (see Data Availability).

### Read Processing and Assemblies

Reads were cleaned of all sequencing primers, duplicate reads were removed, and remaining reads were filtered for quality and length using the CLCBio’s Genomics Workbench (CLCBio, Denmark) (Trim quality score limit = 0.05; maximum number of ambiguities = 2; minimum read length = 40).

We tested three different assembly methods: CLC Genomic Workbench de novo assembler, the Broad Institute’s Trinity assembly pipeline (Grabherr et al. 2011), and Roche’s gsAssembler (Newbler v2.8). The Newbler assembly method was found to be optimal (see supplementary section S1, Supplementary Material online) using the parameters: Seed step = 12, seed length = 16, seed count = 1, minimum overlap length = 40, minimum overlap identity = 90%, alignment identity score = 2, and alignment difference score = −3. We assembled reads from all three tissue-specific libraries separately, as well as combining reads together into a “pooled” assembly. All three tissue assemblies, including the pooled assembly, are available at the NCBI (see Data Availability).

### Comparisons to Core Eukaryotic Gene and Full-Length *S. salar* Sequences

In order to test the quality of our assemblies, we compared them with two different databases. The first comparison gave an estimation of the range of the genes sequenced by comparing the assemblies to the Core Eukaryotic Gene (CEG) database (Parra et al. 2007), using a BLASTx search. A custom python script was used to count the number of unique CEGs hits from the BLASTx search at four different *E*-value thresholds (e^−^^5^, e^−^^20^, e^−^^50^, and e^−^^100^).

The second comparison assessed the proportion of full-length sequences that were present in the assemblies by comparing all four assemblies with a set of full-length *S. salar* sequences (Leong et al. 2010). A BLASTn search was used, and the longest alignment length for each full-length transcript found in the search was retrieved. The BLASTn search was repeated at four different threshold levels (e^−^^5^, e^−^^20^, e^−^^50^, and e^−^^100^).

### Inter-tissue Comparisons and Annotation

Reciprocal BLASTn searches (Overbeek et al. 1999) were carried out between each tissue-specific library in order to assess differences in the sequences found. The longest isotig from each isogroup was used. The searches were carried out and analyzed using a custom python script, and an *E*-value threshold of e^−^^5^ was used.

All four assemblies were compared with a UniProt–SwissProt database, downloaded May 2013 (UniProt Consortium 2012) for functional annotation, using BLASTx (threshold = e^−^^5^). In order to prevent overrepresentation of isogroups with high numbers of isotigs, the BLAST results were filtered so that from each isogroup only the isotig with the lowest *E*-value was retained for further analysis. The BLAST2GO software (Conesa et al. 2005) was then used to map, annotate, and analyze the BLAST results. Enrichment analyses were carried out both between tissue-specific assemblies and the pooled assembly. Fisher’s exact tests were used to test the significance of each result, using a false discovery rate (FDR) threshold of 0.05.

### Comparisons to Salmonid and Other Teleost Sequences

We compared our transcriptome assemblies against several fish databases in order to estimate the amount of novel gene discovery within the lineage. Using BLASTn, comparisons were carried out against four nucleotide databases: The *S. salar* NCBI Unigene database (Pontius and Schuler 2003) (taxon identity [ID] = 8030), and messenger RNA (mRNA) GenBank databases (Benson et al. 2006) for *S. salar*, all salmonid species (taxon ID = 8015) and for all teleosts (taxon ID = 32443). A BLASTn search was used at a range of thresholds (e^−^^5^, e^−^^20^, e^−^^50^, and e^−^^100^). For each isogroup, only the isotig with the lowest *E*-value hit was used for further analysis. For each database, the proportion of isogroups with and without positive BLAST hits was calculated. In addition, comparisons, using BLASTx to salmonid and teleost protein sequences available on the NCBI were carried out, also at a range of *E*-value thresholds (e^−^^5^, e^−^^20^, e^−^^50^, e^−^^100^).

### Duplicate Gene Search

**Fig. 1.—:**
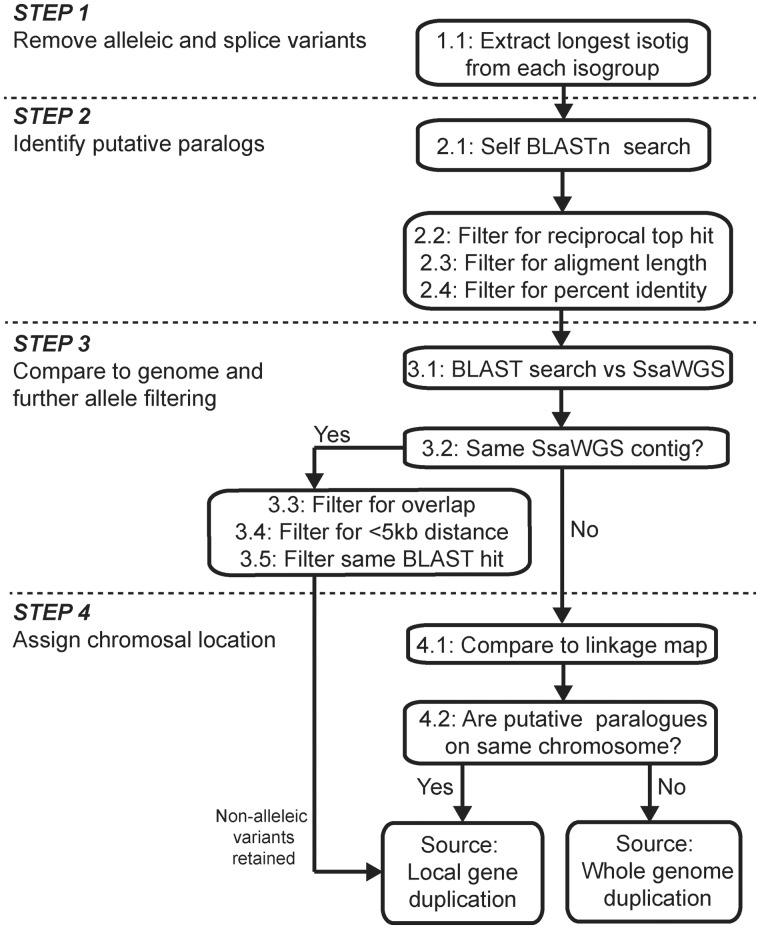
A flow diagram for the method to identify paralog gene pairs and determine whether they originated from a WGD or a LGD event.

We developed a work-flow to identify true duplicate pairs and determine whether they were a result of WGD or more recent, LGD events (fig. 1). First, within gene variants (e.g., different alleles and isoforms) were filtered out from the data set using the clustering of overlapping contigs generated from the Newbler assembly, so that only the longest isotig from each isogroup was used (Step 1, fig. 1). Next, a reciprocal self-BLASTn search within the filtered sequences was performed to identify putative paralog pairs (Step 2.1, fig 4): Sequences were labeled as putative paralog pairs only if they were each other’s top BLAST hit (Step 2.2, fig. 1). The sequences were filtered further, by requiring at least a 300 bp alignment length (Step 2.3, fig. 1) and more than 80% ID along that alignment (Step 2.4, fig. 1), which is a standard benchmark for paralogs in salmonid species (Koop et al. 2008; Leong et al. 2010). Next, possible allelic variants were filtered by BLASTn searching each sequence against the draft *S. salar* whole-genome sequence (SsWGS) (Davidson et al. 2010) (Step 3.1, fig. 1). The genome sequence was downloaded from the NCBI’s website (http://www.ncbi.nlm.nih.gov/genome/369, last accessed June 26, 2014, project ID: 72713). Pairs of sequences that had overlapping hits on the same SsWGS contig were considered allelic variants and discarded from the analysis (Steps 3.2–3.5, fig. 1). If two sequences were found on the same SsWGS contig, were greater than 5 kb apart on the contig, and had different BLAST hits against the *S. salar* Unigene database, they were considered to be paralogs from a LGD (Steps 3.2–3.5, fig. 1). Step 4 assigns chromosomal location to paralogs by BLAST results from the SsWGS that are combined with the linkage map available on www.asalbase.org (last accessed June 26, 2014) (Ng et al. 2005; Danzmann et al. 2008; Moen et al. 2008; Lien et al. 2011) (Step 4.1, fig. 1).

The work flow was implemented using custom python scripts. All BLASTn searches were carried out locally using a threshold of e^−^^20^. We repeated the analysis using percent ID thresholds of 70%, 75%, 80% and 85%, and minimum alignment length thresholds of 300 and 600 bp.

Differences in percent ID between paralog pairs on the same chromosome and those on different chromosomes were tested for using the R-statistics package (v.2.15.3) (R Development Core Team 2012). In order to test whether parametric statistics could be used, the variable percent ID underwent an arc sin transformation (standard for percentage variables), but was shown to be not be normally distributed by a Shapiro–Wilk test, therefore a Mann–Whitney test was used to compare differences.

### Protein Prediction and Ka/Ks Estimations

A tBLASTx search was carried out between each sequence within a paralog pair. The entire sequence of each paralog was translated in the reading frame given by the tBLASTx search. The longest uninterrupted open reading frame that overlapped with the tBLASTx alignment was used for further analysis. The protein sequences were then aligned using CLUSTALw2 (Larkin et al. 2007) and aligned to their DNA sequences using Pal2Nal (Suyama et al. 2006). Ks and Ka/Ks calculations were made using Kaks calculator (Zhang et al. 2006) employing the Nei–Gojobori method (Nei and Gojobori 1986). The resulting alignments were filtered by quality score (minimum score = 100) and manually inspected. For parametric statistical analysis Ka, Ks, and Ka/Ks values were log transformed, and tested for normality using a Shapiro–Wilk test. The tBLASTx searches, protein prediction, CLUSTALW2 alignments, Pal2Nal alignments, and Ka/Ks calculations were executed using a custom made python script (available on DRYAD). Statistical analyses were carried out using the R-statistics package (v.2.15.3) (R Development Core Team 2012).

### Functional Change in Paralogs

Two comparison data sets, C1 (same genes) and C2 (different genes) were generated using a custom python script. For data set C1 the first sequence was chosen at random, and then a second sequence was chosen at random from within the same isogroup. No isogroup, and therefore no related isotigs, were used twice. For data set C2, the first sequence was chosen at random, and then a second sequence was randomly chosen. If the second sequence came from the same isogroup, then it was randomly replaced until a sequence from a different isogroup was found. No sequence was selected twice.

In all three data sets (paralog pairs, C1, and C2), we tested whether each pair within a gene pair had the same BLASTx hit against the salmonid protein database, or whether a different BLAST hit had been retrieved. Sequence pairs were classified as either same BLAST hit, different BLAST hit (including instances where one sequence had a BLAST hit and the other did not), and no BLAST hit. The differences in proportions were tested using a χ^2^ test.

In a second test of functional divergence, we retrieved the gene ontology (GO) terms obtained from the annotation of the transcriptomes for each sequence within a paralog pair. The Jaccard index for the overlap between the set of GO terms was calculated for each paralog pair (Jaccard index is the size of the intersect of two sets divided by the union of the two sets). Significant differences in the Jaccard index between the groups (paralog pairs, C1, and C2) were tested for using Kruskal–Wallis and Mann–Whitney tests.

To further test for potential functional changes, we estimated the coding potential for each sequence using PORTRAIT (Arrial et al. 2009). A PORTRAIT score of less than 0.5 indicates a low probability of that the sequence codes for a protein, and a PORTRAIT score greater than 0.5 indicates a high probability that the sequence codes for a protein. For each pair, we calculated the difference in coding potential. Pairs of sequence that both code for proteins will have a small difference in coding potential and therefore a low difference in PORTRAIT score, similarly two nonprotein-coding sequences will have a small difference in PORTRAIT score. However, if one sequence codes for a protein and the other is noncoding, then a large difference in PORTRAIT score will be seen. To test for differences in median and interquartile ranges (IQR), we ran a permutation test, using 100,000 rounds of resampling. In each round, the differences between the pairwise differences in median were calculated (e.g., the difference in median between each resampled data set was calculated). The probability was calculated by counting how many times the differences in medians from the resampling cycles were greater than the true differences in medians, and then dividing by the number of repetitions. We repeated this process for the IQR. We confirmed the differences in medians using a Mann–Whitney test, with Bonferonni corrections applied for repeated tests (Miller 1981), the advantage of the permutation approach is that we can also test for differences in the dispersal of the data. All processing and combining of data was carried out using python scripts and all statistical analyses described above were carried out using R version 2.15.3 (R Development Core Team 2012). The R-script used for the permutation tests is given in supplementary method S1, Supplementary Material online.

## Results

### Transcriptomic Resources for Tissues Involved in Migration, in *S. salar*

**Fig. 2.—:**
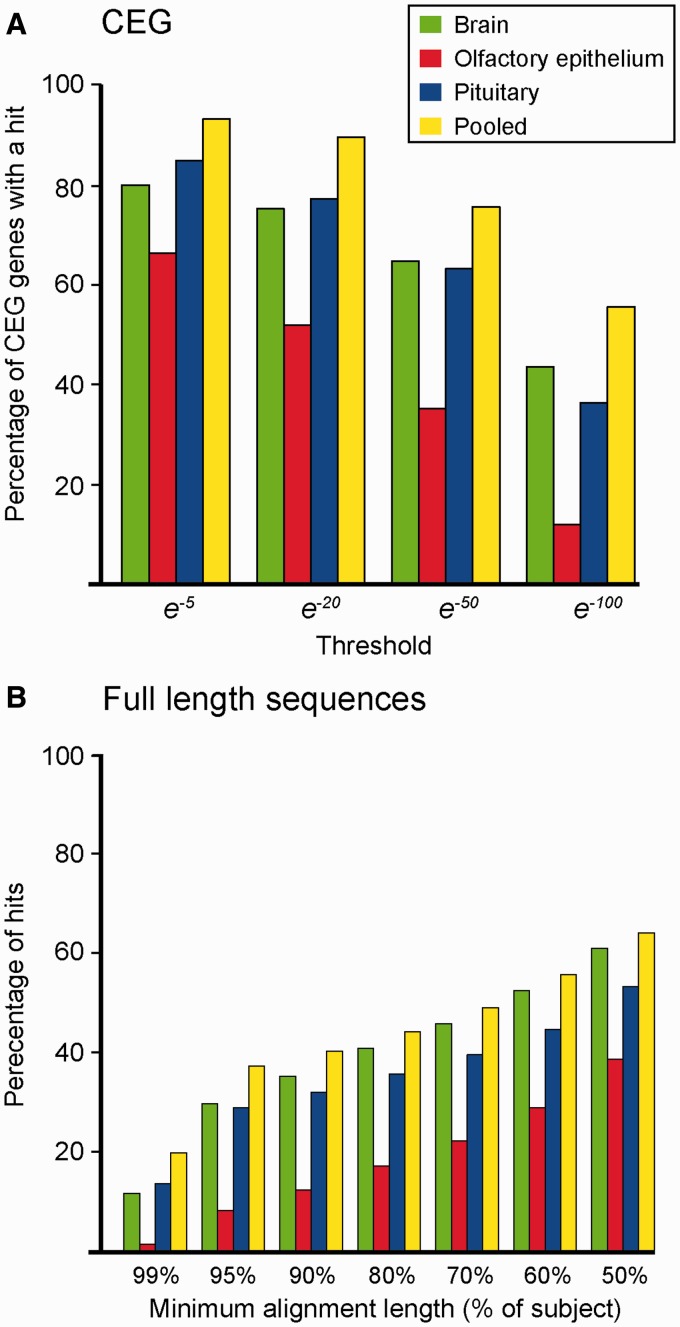
Database comparisons. Each assembly was compared with two databases: (*A*) Using BLASTx, sequences were compared with the CEG database (Parra et al. 2007), which represent a core set of 248 genes expected to be present in all vertebrates at low paralog number (Parra et al. 2007). The proportions of the CEG sequences which were retrieved are given on the *y* axis, at a range of *E*-value thresholds (*x* axis). (*B*) Using BLASTn, sequences were compared with 9,057 full-length *Salmo salar* genes (Leong et al. 2010). Alignment length, given as a proportion of the full-length sequence is given on the *x* axis. The proportion of query sequences above the length on the *x* axis is given on the *y* axis. The data are from analyses performed using an *E*-value threshold of e*^−^*^20^. Tests were carried out at a range of thresholds and the trend was very similar (see supplementary fig. S2.1, Supplementary Material online).

We generated transcriptome sequence assemblies for three different tissues (brain [including the hypothalamus], pituitary gland, and olfactory epithelium), from developing *S. salar* individuals using Roche 454 pyrosequencing technology. The hypothalamus acts as a master regulator of homeostasis and homeorhesis, integrating metabolic, temporal and social inputs (Falcón et al. 2007); the pituitary gland is the source of many important hormones required for progression through PST, such as growth hormone, prolactin, and thyroid stimulating hormone (Prunet et al. 1989; Nordgarden et al. 2007); the olfactory epithelium plays a fundamental role in homing behavior, enabling adult salmon to return to their natal stream (Nevitt and Dittman 1998). The sequencing effort generated over 626 Mb of sequence data (supplementary table S1.1, Supplementary Material online). Using the Newbler assembly program (Roche, Switzerland), the reads were assembled into three tissue-specific transcriptome assemblies, as well as one pooled assembly, which contained between 15,605 and 34,005 isogroups (genes) (table 1; and supplementary section S1, Supplementary Material online, for full details of the assemblies). The transcriptome assemblies were of high quality, containing over 95% of core eukaryotic genes (Parra et al. 2007) (fig. 2*A*) and, when compared with a database of 9,057 of full-length *S. salar* sequences, almost 40% of hits covered greater than 95% of the length of their corresponding BLAST hit (fig. 2*B*; supplementary section S2, Supplementary Material online).

**Table 1:** Sequencing and Assembly Summaries

	Brain	Olfactory epithelium	Pituitary	Pooled
Reads after trimming for primers and quality				
Number of reads	1,013,257	443,891	835,301	2,292,449
Number of nucleotides	296,269,397	118,962,788	211,332,527	626,564,712
Mean read length ± SD (bp)	292 ± 115	268 ± 106	253 ± 104	273 ± 111
Assembly				
Isogroups	16,885	13,162	19,418	34,005
Mean isotig count per isogroup	1.8	1.2	1.5	1.7
Isotigs	23,035	15,605	24,093	45,248
Mean isotig length	1,064 ± 708	683 ± 330	788 ± 456	995 ± 692
Mean contigs per isotig	1.9	1.4	1.6	1.8
Median contig length (lower/upper quartile)	516 (243/868)	526 (324/693)	486 (281/757)	494 (235/840)
Contig N50 (N25/N75)	924 (578/1,439)	650 (467/885)	748 (493/1,100)	905 (558/1,393)
Median contig length (lower/upper quartile)	516 (243/868)	526 (324/693)	486 (281/757)	494 (235/840)
Median read depth per contig (lower/upper quartile)	5 (3.6/8.6)	5.5 (4/9)	5.4 (3.8/8.8)	6.1 (4/11.6)

Note.—Full assembly statistics can be found in supplementary section S1, Supplementary Material online.

#### Tissues Show Significant Transcriptional and Functional Enrichment

**Fig. 3.—:**
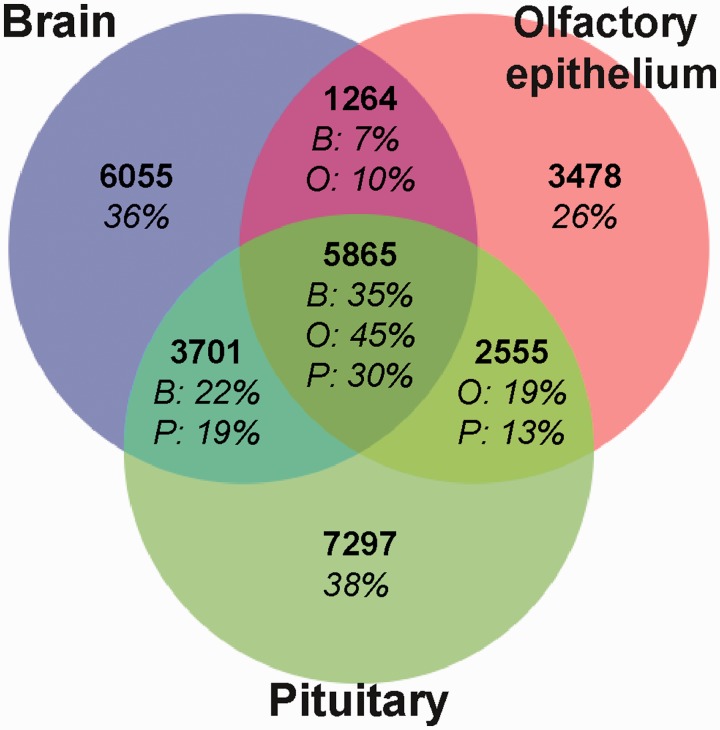
Genes (isogroups) expressed within and among the three tissues, detected using a reciprocal BLASTn search (*E*-value threshold = e^−5^). Only the longest isotig from each isogroup was used. Percentage of isogroups in each assembly is shown. B, brain; O, olfactory epithelium; P, pituitary.

Although there was substantial overlap (62–74%) among isogroups expressed by the three tissue types, a large number of assembled sequences were not shared between all three tissues, indicating distinct differences between the assemblies (fig. 3). Reciprocal BLASTn searches (*E*-value threshold = e^−^^5^) between the assemblies (Overbeek et al. 1999) revealed over 5,800 common isogroups expressed in the three tissues, (fig. 3). However, 26–38% of genes were unique to their assembly, with the olfactory epithelium expressing almost half the number of tissue specific genes as the other two tissues (fig. 3).

Functional analyses revealed that all three tissue types showed equivalent significant functional enrichment when compared with the pooled assembly. The enriched GO terms accurately reflect tissue functions (see supplementary section S3 and data S1, Supplementary Material online). For the brain tissues, 486 GO terms were enriched, including neuronal functional categories, such as axonal cone growth (GO:0001518), voltage-gated sodium channel activity (GO:0005254), and presynaptic membrane (GO:0042734). In the olfactory epithelium, 411 GO terms were enriched. These included receptor complex (GO:0043235) and chemo-attractant activity (GO:0042056), reflecting this tissue’s role in detecting chemical cues in the water. In the pituitary gland, 458 GO terms were enriched. These included hormone activity (GO:0005179), estrogen receptor binding (GO: 0030331), and hormone binding (GO:0042562), reflecting the role of this tissue in hormone production and regulation.

#### New Genes Detected for S. salar

**Fig. 4.—:**
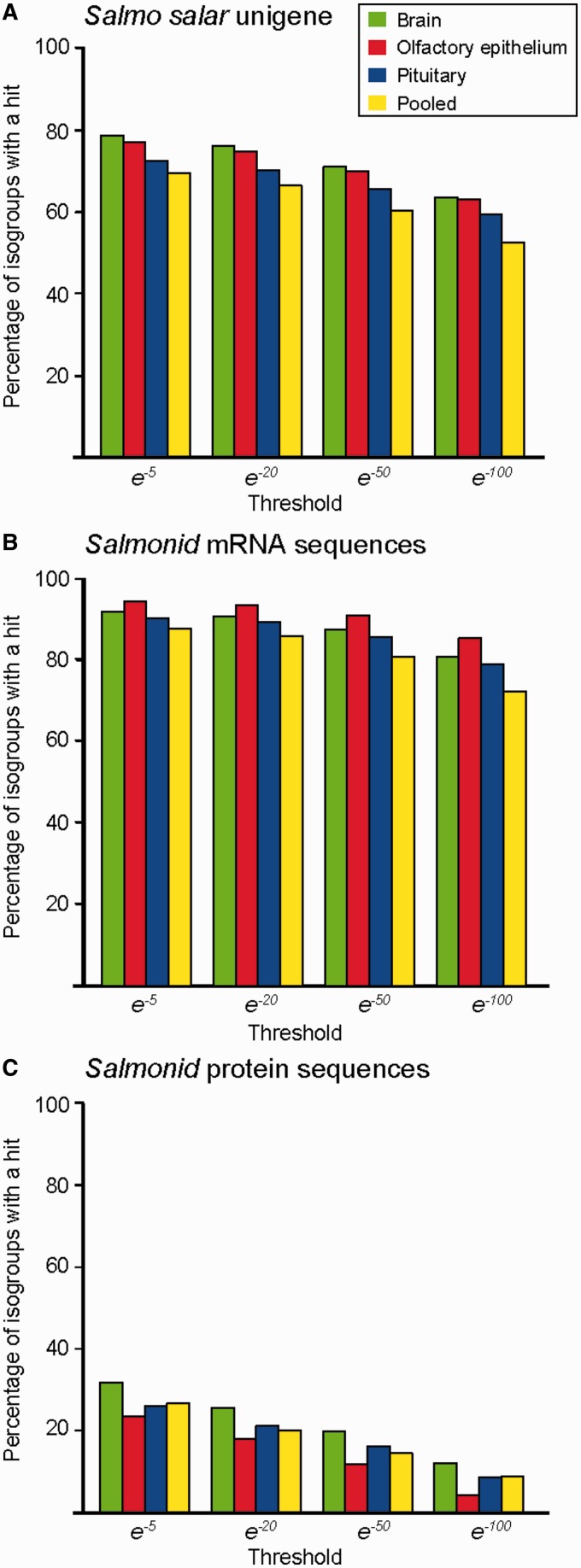
Congruence of genes expressed in each transcriptome assembly with existing genomic resources. (*A*) *Salmo salar* Unigene database (Pontius and Schuler 2003), (*B*) GenBank mRNA database for salmonids (Benson et al. 2006), and (C) NCBI protein database for salmonids (Benson et al. 2006). The percentage of isogroups with significant BLAST hits is given on the *y* axis, and the *E*-value threshold is given on the *x*-axis.

A high proportion (20–50%) of sequences did not have significant matches with the existing *S. salar* Unigene sequences (fig. 4*A*). Even at a relaxed threshold (e^−^^5^), between 22% and 30% (3,634–10,412) of isogroups in each assembly did not have a hit against the *S. salar* database. At more stringent thresholds (>e^−^^50^), the proportion of sequences without hits was nearer 50%. Similar results were found in a comparison with GenBank mRNA *S. salar* sequence data (supplementary fig. S4.1, Supplementary Material online).

The low congruence with available *S. salar* sequence data demonstrates how these important tissues have been undersampled in *S. salar* as we found much higher congruence at more basal taxonomic levels. We compared our assemblies with the GenBank mRNA sequences for the entire salmonid lineage (fig. 4*B*) (Benson et al. 2006), which contains data from over 100 salmonid species including Pacific salmon species (*Oncorhynchus* sp.), grayling (*Coregonus clupeaformis*), and brook trout (*Salvelinus fontinalis*) (fig. 4*B*). Most genes had a significant hit: Only 5–13% of sequences did not have a hit at a relaxed *E*-value threshold (e^−^^5^), and between 20% and 30% did not have hits at more stringent thresholds (>e^−^^50^). As expected, the proportion of positive BLAST hits dropped dramatically when compared with salmonid protein, rather than nucleotide sequence data (fig. 4*C*) between 31.8% and 23.4% (*E-*value threshold = e^−^^5^). We observed a similar trend with the protein database for all teleosts (supplementary fig. S4.1, Supplementary Material online).

### WGD and LGD Events in *S. salar*

The ancestral WGD event at the base of the salmonid lineage has been well studied (Ohno 1970; Allendorf and Thorgaard 1984; Alexandrou et al. 2013; Berthelot et al. 2014; Macqueen and Johnston 2014). However, the level and effects of LGD events after the WGD event are unclear (Koop and Davidson 2008). The first essential step in order to understand this further is to have a reliable method for distinguishing paralog genes from allele- and splice-variants. To do this, we exploited an intrinsic property of the assembly method that identifies and groups potential isoforms and allelic variants to increase the stringency of paralog identification. The second step is to distinguish WGD-derived paralogs from LGD paralogs. For this, we assumed paralogs arising from the WGD event would be located on different chromosomes, whereas those arising from LGD events would located be on the same chromosome. Once we identified paralog origin, we then estimated whether the LGD events were occurring before or after the WGD by comparing the divergence between the two groups.

#### Paralogs Arise through LGD and WGD in S. salar

**Fig. 5.—:**
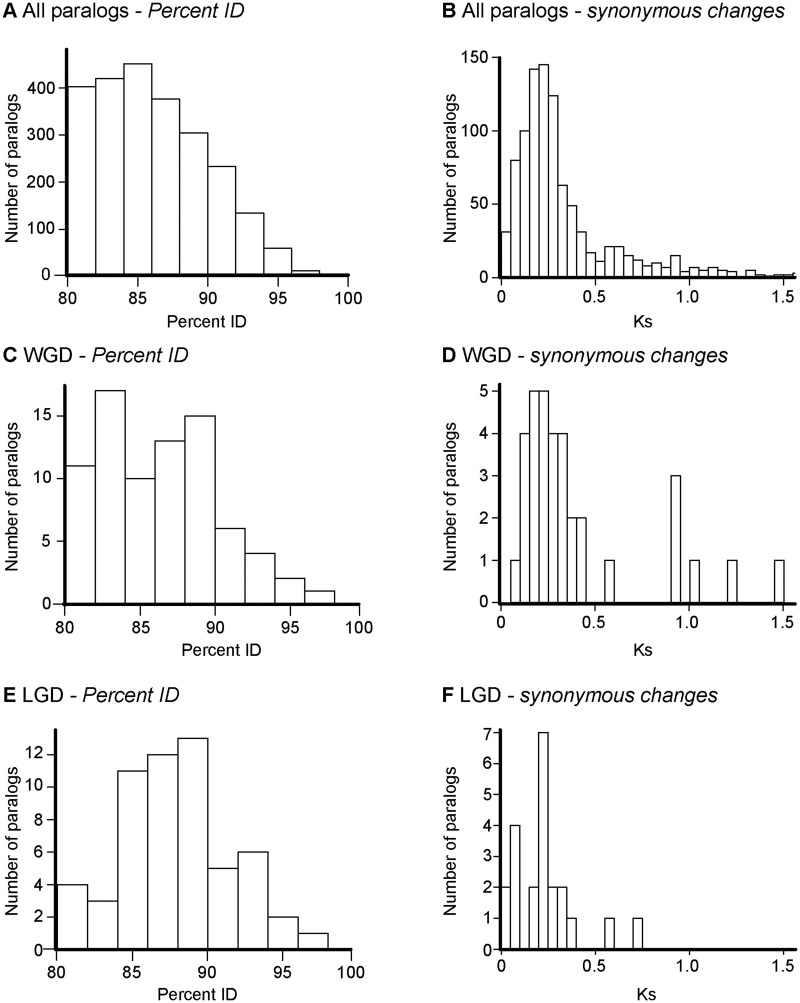
Similarity between paralog sequences in *Salmo salar* expressed in the transcriptomes presented here (minimum percent ID = 80%, minimum alignment length = 300 bp). (*A*, *B*) All 2,394 putative paralog pairs. (*C*, *D*) Paralog pairs where both sequences were assigned to the same chromosome (LGDs). (*E*, *F*) Paralog pairs where the sequences were assigned to the different chromosomes (WGDs). Both percent ID within paralog pairs (*A*, *C*, *E*) and synonymous substitution (Ks) rate (*B*, *D*, *E*) are given. For ease of presentation, Ks values greater than 1.5 are not shown (see supplementary figs. S5.1–S5.8, Supplementary Material online). The analyses were repeated at a range of thresholds (supplementary figs. S5.1–S5.8, Supplementary Material online).

We applied the work flow described in the Materials and Methods and in figure 1, to our pooled transcriptome assembly to identify paralog pairs. In our data set, removal of allelic and splice variants left 33,937 sequences remaining for analysis (Step 1). From these, 2,451 potential paralog pairs were retrieved (Step 2). Fifty-seven paralog pairs were identified as possible allelic variants, and therefore discarded from the analysis (Step 3). The mean percent ID between these paralogs was 86.0% and they clustered between 80% and 90% (fig. 5*A* and *B*). We were able to assign chromosomal locations to both sequences for 135 of the remaining 2,394 duplicates pairs (Step 4). Of these, 79 pairs were located on different chromosomes (fig. 5*C* and *D*), and were likely to arise from the ancestral WGD event, whereas 57 were located on the same chromosome (including one instance where the hits from one paralog pair against the same SsWGS chromosome were judged to be different genes) (fig. 5*E* and *F*).

**Fig. 6.—:**
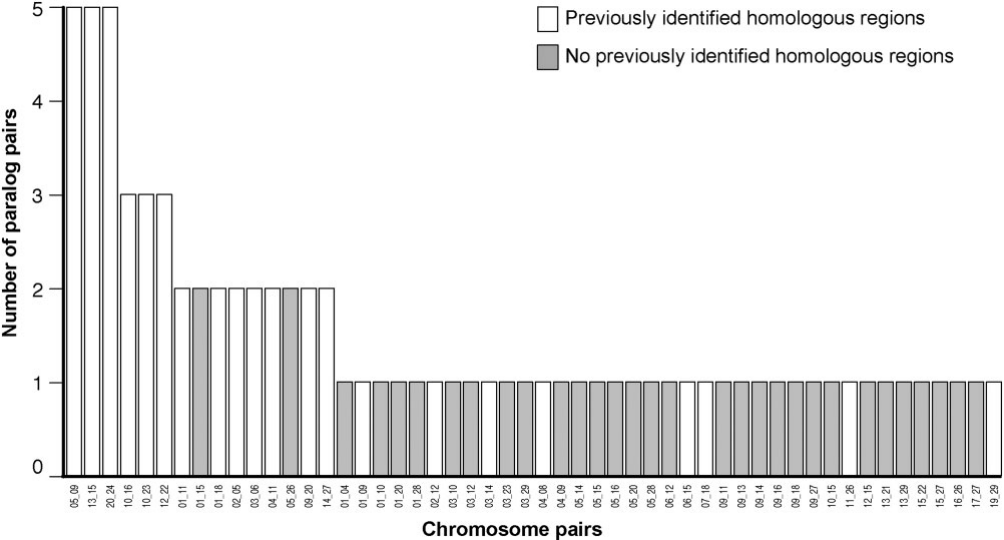
Chromosomal locations of putative paralog pairs compared with previously identified pairs of chromosomes with regions of homology (Phillips et al. 2009; Lien et al. 2011). Chromsome numbers are given on the *x* axis separated by an underscore.

The chromosome locations of the paralog pairs on different chromosomes identified here correspond to previously identified pairs of chromosomes containing homologous regions within the *S. salar* genome (fig. 6) (Phillips et al. 2009; Lien et al. 2011). Twenty-seven pairs of chromosomes that share homologous regions had been identified previously in *S. salar* (Phillips et al. 2009; Lien et al. 2011). Fifty-eight percent (46 out of 79) of the paralog pairs that we identified in our study matched 21 of these 27 (Phillips et al. 2009; Lien et al. 2011) (fig. 6). Furthermore, the six most frequent chromosomal pairings in our study, all matched previously identified chromosomal homologies, with 13 out of the 15 most frequent chromosomal pairings observed here also being identified in previous studies (Phillips et al. 2009; Lien et al. 2011) (fig. 6). The high congruence between our new data set and previous studies provides validation for our approach.

Duplicated pairs on different chromosomes were less similar to each other than those on the same chromosome (86.3% vs. 87.7%; Mann–Whitney *W* = 1,779, *P* = 0.037; fig. 5*A*, *C*, and *E*). In studies addressing specific sets of duplicate genes (e.g., Macqueen et al. 2010, 2013; Rønnestad et al. 2010; Sandbakken et al. 2012), the percent ID between paralogs ranges from less than 70% to over 85%. Similarly, when we looked at synonymous substitution rates, less divergence was observed within pairs on different chromosomes (mean Ks = 0.40, median Ks = 0.27) compared with pairs derived from the same chromosome (mean Ks = 0.23, median Ks = 0.21) (*t*_41.4_ = 2.46, *P* = 0.018). Our results indicate that the LGD events occurred after the WGD. Estimates of divergence times for LGDs ranged from 57–77 to 28–40 Ma (using the upper [∼100 Ma] and lower [∼50 Ma] estimates of the WGD event, respectively, and depending on whether the mean or median Ks was used as a reference). We repeated the whole analysis at different percent IDs and alignment length thresholds, and obtained similar results (supplementary table S5.1 and figs. S5.1–S5.8, Supplementary Material online).

#### No Functional Enrichment among WGD or Local Paralog Pairs

In total, 94 GO terms could be retrieved from the paralog pairs, indicating the broad functionality of genes involved, for example, signaling (GO:0023052), localization (GO:0051179), and response to stimulus (GO:0071840) (see supplementary data S2, Supplementary Material online, for a full list of GO terms). Only one GO term was enriched across all paralog pairs (GO:0006091: generation of precursor metabolites and energy, FDR = 0.00042, corresponding to five paralog pairs), and this enrichment occurred among paralog pairs on the same chromosome. Thus, there appears to be little functional bias in paralogs or between the two types of duplication events (Lukacs et al. 2007).

### Gene Duplication and Changes in Gene Function

We used our full database of the 2,394 paralog pairs to test the hypothesis that there has been functional divergence within paralog pairs. The larger data set was used because it was only possible to provide chromosomal location, and therefore assign WGD or LGD origin, to a small subset of paralog pairs. By using a larger data set we provide a more robust analysis. In addition, all analyses were subsequently carried out on WGD and LGD duplicates separately.

**Fig. 7.—:**
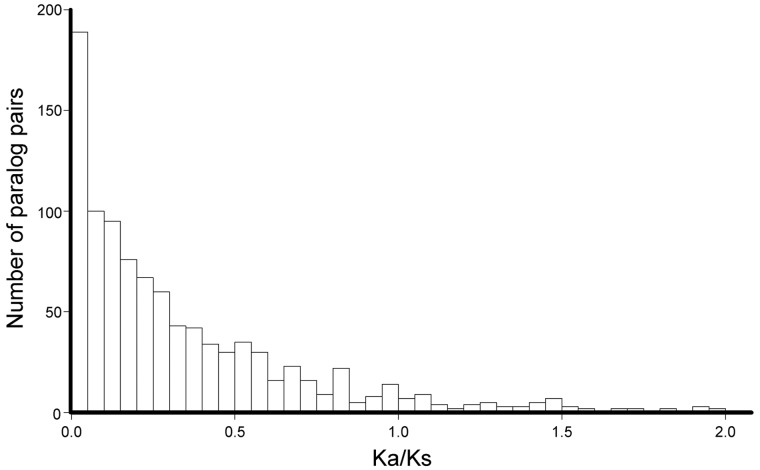
Ka/Ks estimations within paralog pairs. Ka/Ks estimations above 2.0 (*n* = 17), are not shown (maximum = 8.32). See supplementary figure S5.9, Supplementary Material online.

Functional divergence in *S. salar* duplicates has been demonstrated before using Ka/Ks ratios (Leong et al. 2010). Using our sequence alignments, we obtained similar results (fig. 7; median Ka/Ks = 0.21). This confirms a release from purifying selection that would enable functional changes in protein sequence and adaptation. However, we were unable to detect a significant difference in Ka/Ks between WGD and LGD events. We investigated the changes in function further by looking at: 1) Change in the protein-coding function of the duplicate from the ancestral genes, and 2) change of function between protein-coding and nonprotein-coding sequence, such as regulatory long noncoding RNAs (lncRNA).

In order to assess functional divergence, we generated two control data sets of sequence pairs. The first group, control data set 1 (C1: “same genes”), consisted of 3,000 pairs of isotigs taken from the same isogroup in our assembly. This data set would represent splice- and allelic-variants, and only a small amount of functional change, if any would be expected. If little or no functional change was occurring between the paralog pairs, then we would expect to see similar patterns to data set C1. The second group, control data set 2 (C2: “different genes”), consisted of 3,000 pairs of isotigs taken from different isogroups. This second data set represents different genes, and we would expect to see high levels of functional differences within the pairs. In the unlikely event of the paralog pairs completely functionally diverging, we would expect them to look like data set C2. We then tested our paralog pairs against these two data sets: If paralogs are not diverging in function, we would expect them to show similar within-pair functional differences as control data set C1; if functional divergence is occurring we would expect to see similar functional differences as seen in control data set C2.

#### Changes in the Protein-Coding Function of the Duplicate from the Ancestral Genes

**Fig. 8.—:**
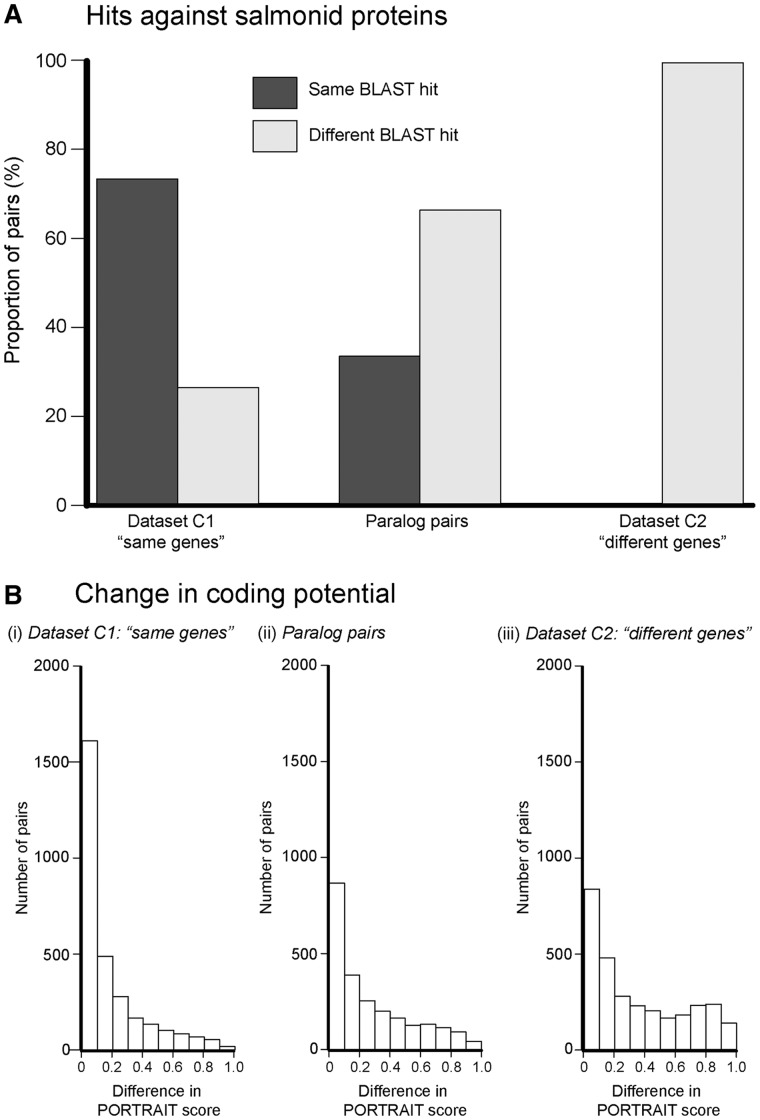
Analyses of functional changes in paralog pairs. (A) BLAST hits for the two control data sets (C1: “same genes” and C2: “different genes”) and the real data set of paralog pairs (identified in WGD and LGD Events in *S. salar*), When compared against an NCBI salmonid protein database. The proportion of pairs is given on the *y* axis. In the majority of sequence pairs, neither sequence had a positive BLASTx hit and they are not shown here (see fig. 4*C*). This analysis was repeated with the salmonid mRNA from GenBank, the *S. salar* Unigene database, and the UniProt protein database, and the same results were obtained (supplementary fig. S6.1, Supplementary Material online). (*B*) The absolute difference in coding potential for each sequence pair across the three data sets (represented by absolute difference within paralog pairs in their respective PORTRAIT scores) (Arrial et al. 2009).

The majority of paralog pair sequences had different functions (fig. 8*A*): Of the 1,119 paralog pairs which had BLASTx hits against the salmonid protein database (*E*-value threshold = e^−^^5^), 33% of pairs had the same BLAST hit, whereas 66% had the different BLAST hits. This pattern was significantly different to what we observed in our two control data sets (χ^2^ = 1,586, df = 4, *P* < 0.0001). As expected, the majority (55%) of data set C1 (same genes) had the same BLAST hit, whereas none of data set C2 (different genes) had the same BLAST hit (fig. 8*A*). The small number of sequences with different hits in data set C1 can be attributed to redundancy within the salmonid protein database, as well as untranslated regions within the sequences altering or disrupting BLAST alignments. We repeated the analysis with the *S. salar* Unigene data set, the salmonid mRNA database and the UniProt database used for annotation (see Tissues Show Significant Transcriptional and Functional Enrichment section), and in all three cases found the same results (supplementary fig. S6.1, Supplementary Material online). For the paralog pairs with different UniProt hits, we retrieved the GO terms for each sequence, and found a significant difference in overlap of GO terms between the paralog pairs and data set C2 (different genes) (*W* = 2,642, *P* < 0.0001) but no significant different between paralog pairs and data set C1 (same genes; supplementary fig. 6.2, Supplementary Material online).

For the both the WGD- and LGD-derived duplicates, significantly different BLAST hits were retrieved (supplementary figs. S7.1–S7.3, Supplementary Material online). However, it was not possible to detect a difference between the proportion of BLAST hits between WGD- and LGD-derived duplicates.

#### Changes in Coding Potential within Paralog Pairs

We detected evidence of changes in coding potential within paralog pairs (fig. 8*B*). For each sequence in all three data sets we estimated the difference in coding potential using PORTRAIT (Arrial et al. 2009) (see Materials and Methods), a value close to 0 indicates a small change in coding potential, whereas a value closer to 1 indicates a large change. Levels of change in protein-coding potential of our paralog pairs were significantly different from data set C1 (where we expect little/no change in coding potential) and data set C2 (where we expect substantial changes in coding potential) in both their medians and IQR (permutation tests: *P* < 0.0001 for all pairwise tests; fig. 8*B*).

As expected, data set C1 (same genes) sequence pairs had the lowest and most tightly clustered difference in coding potential (median = 0.08, IQR = 0.22), with only 11% had a difference of greater than 0.5. Data set C2 (different genes) had the largest proportion of genes with a difference in coding potential with a greater spread of differences compared with the data set C1 (median = 0.26, IQR = 0.54), as expected if coding genes are paired randomly with noncoding sequences. The paralog pairs showed an intermediate median (0.18) and IQR (0.39). The significantly larger spread (IQR) of differences in PORTRAIT score compared with data set C1, as shown by the differences in IQR, demonstrates that functional changes between coding and noncoding function are occurring within the paralog pairs. However, the paralog pairs do not overlap totally with data set C2, indicating that paralogs still overlap functionally. When comparing the WGD and LGD events separately, significant differences in PORTRAIT score (both median and IQR) occurred within paralog pairs for both types of duplication event (supplementary figs. S7.1 and S7.2, Supplementary Material online). However, no significant differences were detected between the two groups (supplementary fig. S7.3, Supplementary Material online).

Three paralog pairs indicate that genome duplication may be involved in generating functional diversity in olfactory receptors, which may aid evolution of the anadromous life-cycle. In the first pair (isotig42427/isotig42773), both sequences have BLASTx hits against the same protein: Olfactory receptor family C subfamily 4 member 11 (GenBank accession: ACM08808.1). In the second and third paralog pairs (isotig32594/isotig38278 and isotig32202/isotig38751), one sequence from the pair had a hit against olfactory receptor family C subfamily 4 member 11 (GenBank accession: ACM08808.1) but the other sequence retrieved no BLAST hits at all, despite high estimated coding potentials (0.48–0.95).

## Discussion

### Transcriptomic Resources for *S. salar*

Here, we report the transcriptomes for three tissues which have pivotal roles in regulating migration and phenotypic plasticity in *S. salar*. This effort has generated a substantial database of novel sequence data for these tissues as well as for *S. salar* itself. The high number of hits against the CEG database and high coverage of full-length *S. salar* sequences indicate these sequence data presented here are of high quality and cover a high number of sequences in the transcriptome.

Understanding the genomic basis of the anadromous life-cycle of *S. salar* is important both economically and culturally, but also from an evolutionary point of view in understanding how such plastic life-histories arise from a shared genome. The brain, olfactory epithelium, and pituitary gland are instrumental organs in coordinating and executing the metamorphic transitions and behaviors required for migration. The low number of hits from our sequences against current salmonid databases indicates that these tissues are undersampled. Hence, for understanding how migration is coordinated and how it evolves, our transcriptomes will provide important resources for future study. The large amount of novel sequence data here will also aid the assembly and annotation of the *S. salar* genome, which is currently underway (Davidson et al. 2010). Due to the ancestral WGD in the salmonid lineage, genome assembly and annotation is challenging, but some problems (e.g., gene identification) can be ameliorated with an increased knowledge of which sections of the genome are being expressed.

### A High Number of Paralogs in *S. salar* Arise from WGD and LGD Events

It was previously proposed that a high number of LGD events occurred after the ancestral salmonid WGD event (Koop and Davidson 2008). Our study provides a more comprehensive study of this and confirms the hypothesis. Further, it indicates that paralogs occurring from LGD events are occurring at a high rate (∼42% of paralogs detected). Interestingly, this rate is lower than previously suggested (Koop and Davidson 2008). Our study provides important methodological advances by filtering for allelic and splice-variants and assigning genomic location. Thus, our method provides a more accurate estimate of the relative prevalence of WGD- and LGD-derived paralogs.

Our analyses indicate that the LGD events were taking place after the WGD events, as predicted in (Koop and Davidson 2008). The percent ID difference between WGD and LGD differences was very small (∼1%), indicating that the LGD events are occurring soon after the WGD events. Using the Ks estimations, we estimated the LGD events to be occurring between 10 and 40 Myr after the WGD event. The differences in Ks and percent ID could be due to differences in the types of sequences sample. Rates of divergence using Ks are only applicable in protein-coding sequences, and in the recently released rainbow trout genome a higher degree of percent ID divergence between protein-coding sequences than nonprotein-coding sequences was found (Berthelot et al. 2014). Indeed, if we compared percent ID between LGD and WGD using only pairs where it was possible to make Ks estimations, then the difference between the two groups increases to almost 2%. Another difficulty in estimating the relative timing of the two types of duplication events is that, although WGD paralogs will arise at the same time, the LGD events will be happening over a wider timescale. Evidence from the Chinook salmon, *Oncorynchus tshawyscha*, indicates that LGD events have happened very recently, although the exact timing of this is not clear (Brieuc et al. 2014).

A WGD provides an amenable environment for LGD events to occur. First, as part of the transition from a tetraploid to a diploid genome, recombination site misalignment will be occurring causing local duplication of genes. Second, WGD events create a large amount of redundant sequence, which leaves the genome more vulnerable to invasion by new transposons. The activity of transposons may be important in LGDs (Hurles 2004) as they can move around the genome and the inverted repeat sequences which flank them can interfere with recombination. Transposon activity in *S. salar* has been substantial since the WGD event, and includes lateral transfer from parasites (de Boer et al. 2007). Once the genome has reverted to a diploid state, transposon activity will decrease and erroneous recombination is less likely (de Boer et al. 2007), resulting in less favorable conditions for LGD events.

Future research should focus on expanding and building upon our method for conservative detection of paralogs. In order to ensure accuracy in our paralog identification, we restricted ourselves to the top reciprocal BLASTn hits within our transcriptomes. This created two limitations. First, if a LGD paralog and a WGD paralog were present for a gene, then the latter paralog would be missed in our method. Second, if the paralogs had diverged in expression pattern, with one of the paralogs losing expression in our sampled tissues sample (Singh and Hannenhalli 2008), then again the paralog pair would not be detected. Both of these issues can be resolved with wider transcriptome sampling, along with gene prediction algorithms using the salmon genome sequence. The current genome sequence available for *S. salar* is highly fragmented (555,960 contigs, N50 = 9,342 bp), and thus prevented such an approach on a multigene level. Our approach of using transcriptome sequence therefore currently provides the best method for genome wide paralog prediction.

Genome-wide approaches such as ours will be complemented by studying specific gene families*,* as has been done in leptins (Rønnestad et al. 2010), akirins (Macqueen et al. 2010), insulin-like growth factor binding proteins (Macqueen et al. 2013), and melanopsins (Sandbakken et al. 2012). Such approaches can look at the fine details of gene duplication in individual case, which is beyond the scope of our genome-wide approach.

Finally, comparisons between different salmonid species will shed light onto how differences in genomic architecture, environment and selection pressures effect processes after a WGD event, including the generation and fate of paralog pairs (Zhu et al. 2012). For example, it is well established that the WGD event caused changes in genome structure among salmonids (Lien et al. 2011; Phillips et al. 2013), although the reasons behind the variation and its effects on paralog creation and preservation are unclear. We hypothesize that transposable elements are involved in LGD events in *S. salar*; it has been shown that post-WGD transposable element activity is highly genome specific in *Brassica* (Sarilar et al. 2013). LGD events have been detected in both trout (Berthelot et al. 2014) and chinook salmon (Brieuc et al. 2014), but their prevalence is unclear.

### Functional Divergence among Duplicates

Our analyses revealed that paralog genes in *S. salar* show evidence of diverging protein-coding function (fig. 8*A*) including changes in protein-coding function and nonprotein-coding roles (fig. 8*B*). After duplication events, paralogs may become fixed in function (e.g., for maintaining correct dosage levels), or can change in function (either through subfunctionalization or neofunctionalization). The resources available to studying salmonid gene function make it difficult to differentiate between subfunctionalization and neofunctionalization. Our results suggest that functional divergence is occurring, but that sequences within paralog pairs still maintain a large amount of overlap. Thus, subfunctionalization maybe occurring via temporal and/or spatial expression pattern variation.

Previous studies on the fate of paralog sequences predominantly focus on genes with protein-coding function. However, noncoding RNAs are important in modulation of chromatin structure and transcriptional regulation, and thus hold great potential for generating phenotypic plasticity through differential expression of shared genes (Lu et al. 2012; Zhu et al. 2012). Here, we consider possible changes in the functions of both protein-coding and nonprotein-coding sequences. We found that a large number of paralogs diverging between coding and noncoding RNA (fig. 8*B*). Such changes in molecular function and the regulatory machinery in these tissues are likely to generate substantial phenotypic plasticity from the same genome, facilitating the evolution of the anadromous life history in certain salmonid groups. Understanding the role of these noncoding genes in the evolution and regulation of the plastic life history in salmon would be an important future direction.

We have provided some enticing preliminary evidence that such gene duplications are involved in generating diversification of the olfactory receptors. Olfaction is essential for enabling *S. salar* to return to their natal stream (Hasler et al. 1978; Dukes et al. 2004), and therefore diversification in the olfactory receptors is expected (Johnstone et al. 2009, 2012). Our study provides some evidence that this may have been facilitated by genome duplications. A high number of olfactory receptors have already been identified in the *S. salar* genome, but this may in fact be an under estimation (Johnstone et al. 2009, 2012).

An interesting question would be whether the changes in function between LGD- and WGD-derived paralogs were different. It is expected that paralogs on different chromosomes would have more divergent regulation compared with paralogs from LGD events and theoretical models show that paralogs in close linkage will diverge in function (Bozorgmehr 2012). This is supported by evidence in several plant species, where WGD paralogs are associated with subfunctionalization through divergent expression, whereas higher rates of relaxed purifying selection and novel gene function (neofunctionalization) have been observed (Guan et al. 2007; Carretero-Paulet and Fares 2012; Fares et al. 2013). Our analysis was limited by the available linkage map and genome sequence for *S. salar.* We were only able to allocate a chromosomal position in about 4% of the whole-genome contigs (supplementary table S5.1, Supplementary Material online) and as result may have not been able to sample a sufficient number of paralogs to detect any differences.

Comparisons between a wider range of salmon species will give further insight into how such a dramatic anadromous life-cycle evolved, and how WGD and LGD events contributed to it. Recent evidence suggests that anadromous life histories may have arisen independently in the Atlantic (*S. salar*) and Pacific (*Oncorhynchus* sp) salmon species (Alexandrou et al. 2013). Comparisons between the two lineages would provide an ideal opportunity to determine the importance of whole genome and LGDs in enabling individuals to adapt to an anadromous life history. Evidence from *Saccharomyces cerevisiae* suggests that WGD can be very important for adaptation of new life-history strategies, as paralogs created from a WGD event were shown to be recruited for the fermentation process, therefore used for adaption to a novel environment (Chen et al. 2008).

Genome duplications have been proposed as important evolutionary events as they provide a source of redundant genetic material that may facilitate adaptation to a broad range of environments and evolution of life-history strategies. Indeed, two genome duplications have occurred at the base of the vertebrate lineage and are thought to be pivotal to the group’s radiation (Dehal and Boore 2005), as well as the numerous genome duplications observed in plants that are key to their evolution (Sémon and Wolfe 2007). The results presented here highlight one case study*, S. salar*, and demonstrate that a WGD followed by multiple LGD events, can generate a large amount of divergent genetic material that may facilitate adaptation. Our study investigates the nature and framework of duplication events and provides a springboard for testing the hypothesis that WGD and LGD events have enabled *S. salar* to evolve the discrete phenotypic variability required to adapt to their anadromous life history.

## Supplementary Material

Supplementary file S1, data S1 and S2, and method S1 are available at *Genome Biology and Evolution* online (http://www.gbe.oxfordjournals.org/).

Supplementary Data
