# Supplementary material for: Extensive Local Gene Duplication and Functional Divergence among Paralogs in Atlantic Salmon
Source: Genome Biol Evol. 2014 Jun 19;6(7):1790–805. doi: 10.1093/gbe/evu131 (PMC4122929; doi:10.1093/gbe/evu131)
Supplement: Supplementary Data [file supp_6_7_1790__index.html]

Extensive local gene duplication and functional divergence among paralogs in Atlantic salmon — Extensive Local Gene Duplication and Functional Divergence among Paralogs in Atlantic Salmon — Supplementary Data 

# Extensive Local Gene Duplication and Functional Divergence among Paralogs in Atlantic Salmon

## Supplementary Data

files

**Files in this Data Supplement:**

- Supplementary Data - zip file
