# Supplementary material for: Extensive Local Gene Duplication and Functional Divergence among Paralogs in Atlantic Salmon
Source: Genome Biol Evol. 2014 Jun 19;6(7):1790–805. doi: 10.1093/gbe/evu131 (PMC4122929; doi:10.1093/gbe/evu131)
Supplement: Supplementary Data [file supp_evu131_suppl_data.zip › suppfile1.docx]

**Supplementary Section 1: Further details on sequencing results, assembly and annotation**

*S1.1 Sequencing results and cleaning*

We extracted RNA from three different tissues from developing *Salmo salar* individuals: the brain (including the hypothalamus), pituitary gland and olfactory epithelium. Tissue-specific cDNA libraries were sequenced using Roche 454 pyro-sequencing, with one plate per tissue. This resulted in between 805,627 and 1,305,805 reads containing between 169 mega-base pairs (Mbp) and 375Mbp of data for each library (Table SI 1.1). Reads were filtered and trimmed for quality, and adapter sequences were removed which left between 443,891 to 1,013,257 reads remaining (Table SI1).

*S1.2 Assembly trials*

We tested three methods of assembly: First, we used the CLC Genomic Workbench *de novo* assembler under a variety of parameters. Second, the Broad Institute’s Trinity Assembly pipeline (Grabherr et al. 2011) was used using the following parameters (--JM 90G --SS_lib_type F --min_kmer_cov 2). Third, we used Roche’s gsAssembler (Newbler v2.8), again several parameter combinations were tested. A Newbler assembly was found to be best due to increased contig length and coverage (parameters: seed step = 12, seed length = 16, seed count = 1, minimum overlap length = 40, minimum overlap identity =90%, alignment identity score = 2, alignment difference score = -3) (Table SI2-5). The latter produced the best assembly due to longer reads, with greater coverage. Furthermore, Newbler groups contigs that consist of alternative arrangements of subsets of reads into isogroups (Anon 2011), therefore reducing redundancy without losing information (Supplementary file 1 and see below). Since we sampled across a range of tissues, individuals, and developmental time points we would expect to detect a high number of allelic and splice variants. All subsequent analyses were therefore conducted on the Newbler assemblies.

*S1.3 Newbler assembly*

We generated four assemblies: three tissue-specific assemblies and one combined assembly of all three tissues (referred to as “pooled”). The number of contigs generated, along with their length and depth reflected the number of reads going into the assembly, with the brain producing the longest sequences and highest coverage among the individual tissue libraries (Table 6).

Newbler accounts for sequence variation (e. g., spice variants) by clustering groups of overlapping contigs (‘exons’) into isotigs (‘isoforms’), which are then grouped into isogroups (‘genes’) (Anon 2011) (and see (Choi et al. 2010)). After clustering, there were between 15,605 and 34,005 genes per tissue-specific assembly (Table 6), with an average of between 1.2 and 1.8 isoforms per isogroup. This is a skewed distribution with between 67% and 86% of genes containing a single contig. There were 34,005 genes in the pooled assembly; the total gene count for *S. salar* is expected to be around 30,000 (Davidson et al. 2010). We expect there to be a degree of redundancy within isogroups due to some allelic variance, as well as punctuated sequencing of gene sequences, which would result in the reads originating from different parts of the same gene being assigned to different isogroups

*Table S1.1: Sequencing results*

|  | **Brain** | **Olfactory epithelium** | **Pituitary** | **Pooled** |
| --- | --- | --- | --- | --- |
| ***Raw Reads*** |  |  |  |  |
| Number of reads | 1,305,805 | 805,627 | 1,027,770 | 3,139,202 |
| Number of nucleotides | 375,504,279 | 169,368,356 | 273,660,814 | 818,533,449 |
| Mean read length ± s.d. (bp) |  ±  |  ±  |  ±  |  ± 152.42 |
|  |  |  |  |  |
| ***Reads after trimming for primers and quality*** |  |  |  |  |
| Number of reads | 1,013,257 | 443,891 | 835,301 | 2,292,449 |
| Number of nucleotides | 296,269,397 | 118,962,788 | 211,332,527 | 626,564,712 |
| Mean read length ± s.d. (bp) | 292 ± 115 | 268 ± 106 |  ±  |  ±  |

*Table S1.2: Assembly trials for brain tissues*

| **Assembly** | **CLC** | **Trinity** | **Newbler** |
| --- | --- | --- | --- |
| Number of contigs | 54,048 | 50,663 | 29,635 |
| Mean contig length ± s.d. (bp) | 562 ± 5 | 450 ±  |  ±  |
| Median contig length (Lower/upper quartile) | 562 (296/664) | 351 (260/508) | 516 (243/868) |
| Contig N50 (N25/N75) | 669 (439/1176) | 485 (325/823) | 924 (578/1439) |
| Mean reads per contig ± s.d. | 14.0 ± 113.7 | 13.1 ±  | 28.80 ±  |
| Median reads per contig (Lower/upper quartile) | 4 (2/9) | 8 (5/13) | 10 (6/22) |
| Mean read depth per contig ± s.d. | 5.2 ± 5 | 5.9 ± 12.9 | 13.9 |
| Median read depth per contig (Lower/upper quartile) | 2.4 ±  | 4.8 (3.0/6.7) | 5.0 (3.6/8.6) |
| Number of singletons | 256,045 | 148,219 | 171,989 |

*Table S1.3: Assembly trials for olfactory epithelium reads*

| **Assembly** | **CLC** | **Trinity** | **Newbler** |
| --- | --- | --- | --- |
| Number of contigs | 34,518 | 25715 | 18,007 |
| Mean contig length ± s.d. (bp) | 466 ± 6 |  ±  |  ±  |
| Median contig length (Lower/upper quartile) | 402 (288/569) | 344 (261/467) | 526 (324/693) |
| Contig N50 (N25/N75) | 529 (378/792) | 419 (307/591) | 650 (467/885) |
| Mean reads per contig ± s.d. | 9.1 ±  | 13.5 ± 15.4 | 17.9 ± 21.0 |
| Median reads per contig (Lower/upper quartile) | 4 (3/9) | 9 (6/16) | 11 (7/20) |
| Mean read depth per contig ± s.d. | 4.3 ±  |  ±  | 8.0 |
| Median read depth per contig (Lower/upper quartile) | 2.6 (1.8/4.4) | 5.4 (3.6/8.1) | 5.5 (4.0/9.0) |
| Number of singletons | 129,190 | 100,928 | 110,272 |

*Table S1.4: Assembly trials for pituitary reads*

| **Assembly** | **CLC** | **Trinity** | **Newbler** |
| --- | --- | --- | --- |
| Number of contigs | 57,817 | 46,614 | 28,329 |
| Mean contig length ± s.d. (bp) | 484 ± 40 | 319 ±  |  ±  |
| Median contig length (Lower/upper quartile) | 397 (267/582) | 319 (249/451) | 486 (281/757) |
| Contig N50 (N25/N75) | 567 (385/936) | 410 (290/636) | 748 (493/1100) |
| Mean reads per contig ± s.d. | 10.8 ± 6 | 12.4 ± 4 | 22.1 ± 2.0 |
| Median reads per contig (Lower/upper quartile) | 4 (3/9) | 9 (5/14) | 11 (7/24) |
| Mean read depth per contig ± s.d. | 4.5 ±  | 6.0 ±  | 8.8 |
| Median read depth per contig (Lower/upper quartile) | 2.5 (.8/4.3) | 5.2 (3.3/7.4) | 5.4 (3.8/8.8) |
| Number of singletons | 210,299 | 192,000 | 187,276 |

*Table S1.5: Assembly trials for all reads pooled together*

| **Assembly** | **CLC** | **Newbler** |
| --- | --- | --- |
| Number of contigs | 102,592 | 58,557 |
| Mean contig length ± s.d. (bp) | 506 ± 7 |  ±  |
| Median contig length (Lower/upper quartile) | 394 (252/594) | 494 (235/840) |
| Contig N50 (N25/N75) | 612 (395/1117) | 905 (558/1393) |
| Mean reads per contig ± s.d. |  ±  | 9 ±  |
| Median reads per contig (Lower/upper quartile) | 5 (3/17) | 12 (6/31) |
| Mean read depth per contig ± s.d. | 6.5 ± 4 | 14.8 |
| Median read depth per contig (Lower/upper quartile) | 2.8 (1.9/5.5) | 6.1 (4.0/11.6) |
| Number of singletons | 545,307 | 292,829 |

*Table S1.6: Full details of Newbler assemblies.*

|  | **Brain** | **Olfactory epithelium** | **Pituitary** | **Pooled** |
| --- | --- | --- | --- | --- |
| ***Assembly*** |  |  |  |  |
| Number of contigs | 29,635 | 18,007 | 28,329 | 58,557 |
| Mean contig length ± s.d. (bp) |  ±  |  ±  |  ±  |  ±  |
| Median contig length (Lower/upper quartile) | 516 (243/868) | 526 (324/693) | 486 (281/757) | 494 (235/840) |
| Contig N50 (N25/N75) | 924 (578/1439) | 650 (467/885) | 748 (493/1100) | 905 (558/1393) |
| Mean reads per contig | 28.0 ±  | 17.9 ± 21.0 | 22.1 ± 2.0 | 49 ±  |
| Median reads per contig (Lower/upper quartile) | 10 (6/22) | 11 (7/20) | 11 (7/24) | 12 (6/31) |
| Mean read depth per contig | 13.9 | 8.0 | 8.8 | 14.8 |
| Median read depth per contig (Lower/upper quartile) | 5 (3.6/8.6) | 5.5 (4.0/9.0) | 5.4 (3.8/8.8) | 6.1 (4.0/11.6) |
| Contig GC Content | 43.6% | 40.6% | 41.2% | 41.7% |
| Number of singletons | 171,989 | 110,272 | 187,276 | 292,829 |
| Mean singleton length ± s.d. (bp) |  ±  |  ±  |  ±  |  ±  |
| Median singleton length (Lower/upper quartile) | 289 (184/376) | 273 (171/342) | 244 (159/319) | 261 (89.5/309) |
| Singleton GC Content | 43.1% | 41.4% | 42.3% | 42.9% |
|  |  |  |  |  |
| IsoGroups | 16,885 | 13,162 | 19,418 | 34,005 |
| Mean isotig count per isogroup | 1.8 | 1.2 | 1.5 | 1.7 |
| Isotigs | 23,035 | 15,605 | 24,093 | 45,248 |
| Mean isotig length |  ±  |  ±  |  ±  |  ±  |
| Mean contigs per isotig | 1.9 | 1.4 | 1.6 | 1.8 |

**Supplementary Section 2: Comparisons of assemblies to full-length *S. salar* sequences (Leong et al 2010)**


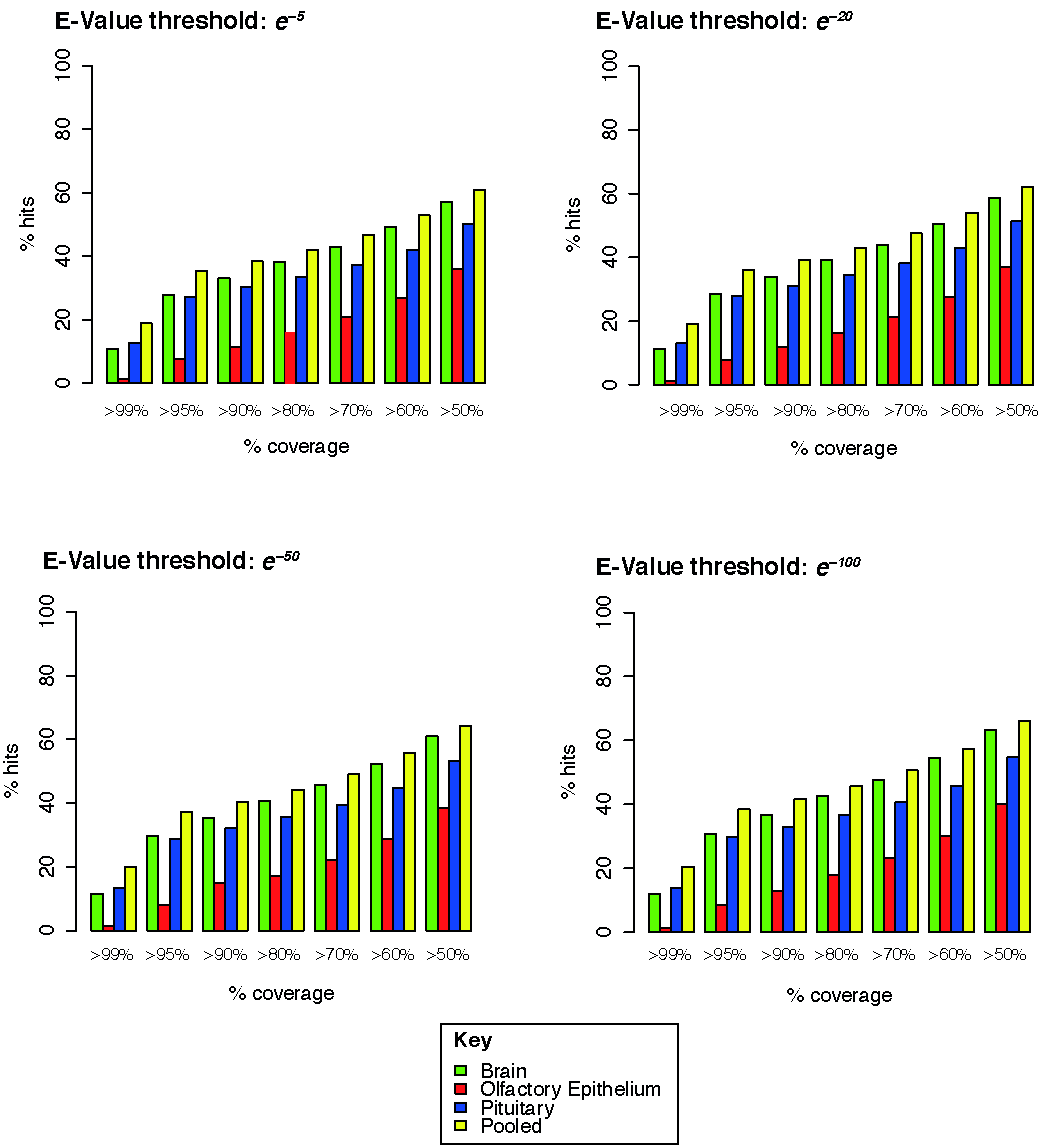


*Figure S2.1:* Using BLASTn, sequences were compared to 9,057 full-length *S. salar* genes (Leong et al. 2010). Alignment length, given as a proportion of the full-length sequence is given on the x-axis. The proportion of query sequences above the length on the x-axis is given on the y-axis. The data is from analyses performed using an E-value thresholds of *e^-5^* to *e^-100^*. Only the isotig with the lowest E-value hit was used from each isogroup.

**Supplementary Section 3: Annotation**

In order to obtain functional information, all four assemblies were compared against a UniProt-SwissProt database (Camon et al. 2004; The UniProt Consortium 2012). At an E-value threshold of *e^-5^*, a large number of annotations covering a range of GO terms were retrieved for each assembly that covered a range of function (Fig. S1.1; hypothalamus: 4,986 unique GO terms from 27,364 annotations; olfactory rosette: 3638 unique GO terms from 17,116 annotations; pituitary gland: 5,108 unique GO terms from 26,334 annotations; pooled: 6,897 GO terms from 51,422 annotations).


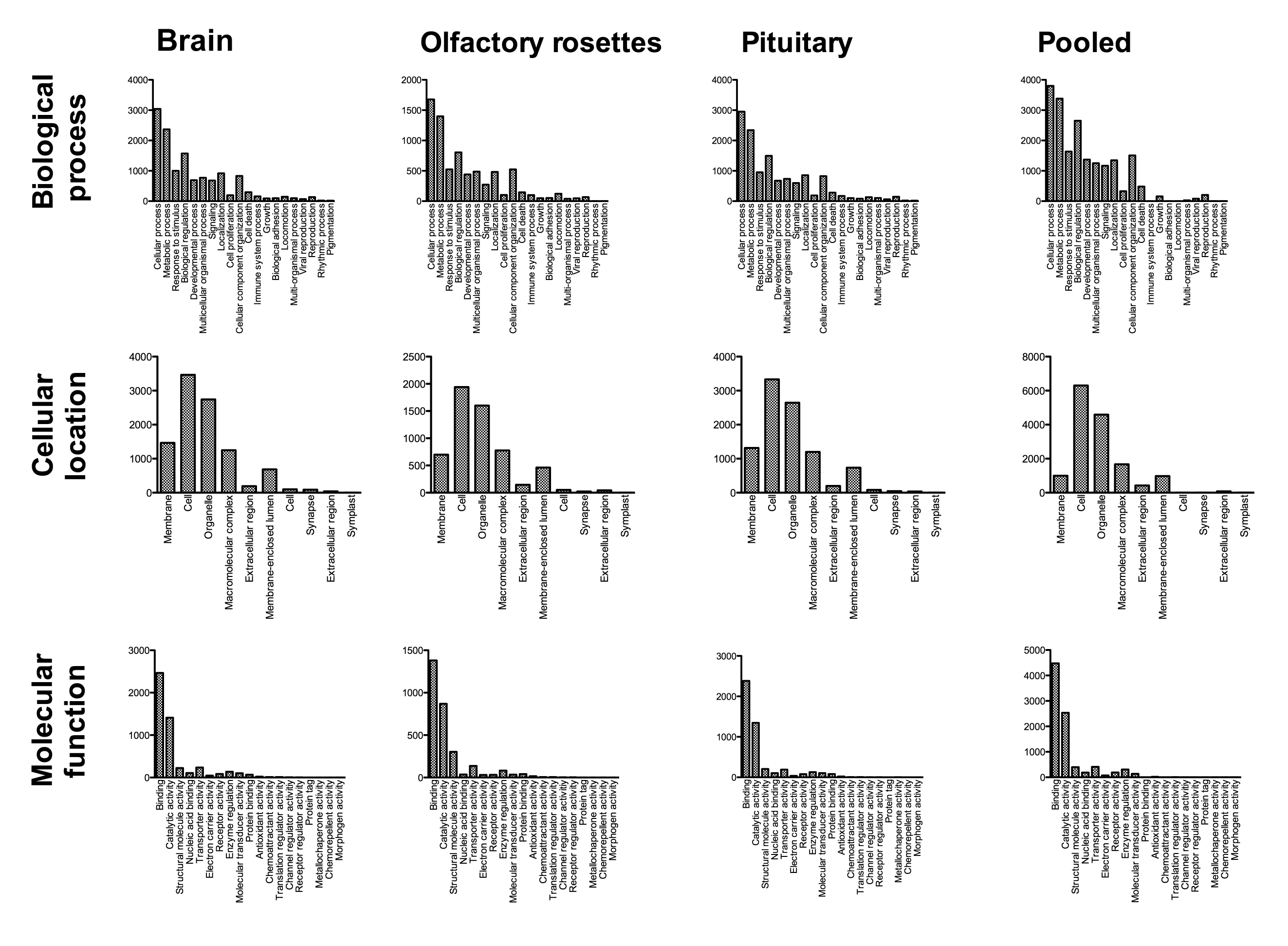


*Figure S3.1* Level two GO terms for the four assemblies divided into biological process, cellular location, and molecular function. Due to the large amount of sequences and annotations retrieved, a seq filter of 10 was required for creating GO maps for biological process terms for the brain, olfactory rosette and, pituitary, and a seq filter of 20 was used for all GO maps for the pooled assembly (Conesa et al. 2005).

**Supplementary Section 4: Transcriptome assembly comparisons to *S. salar* mRNA and Teleost non-redundant protein database.**


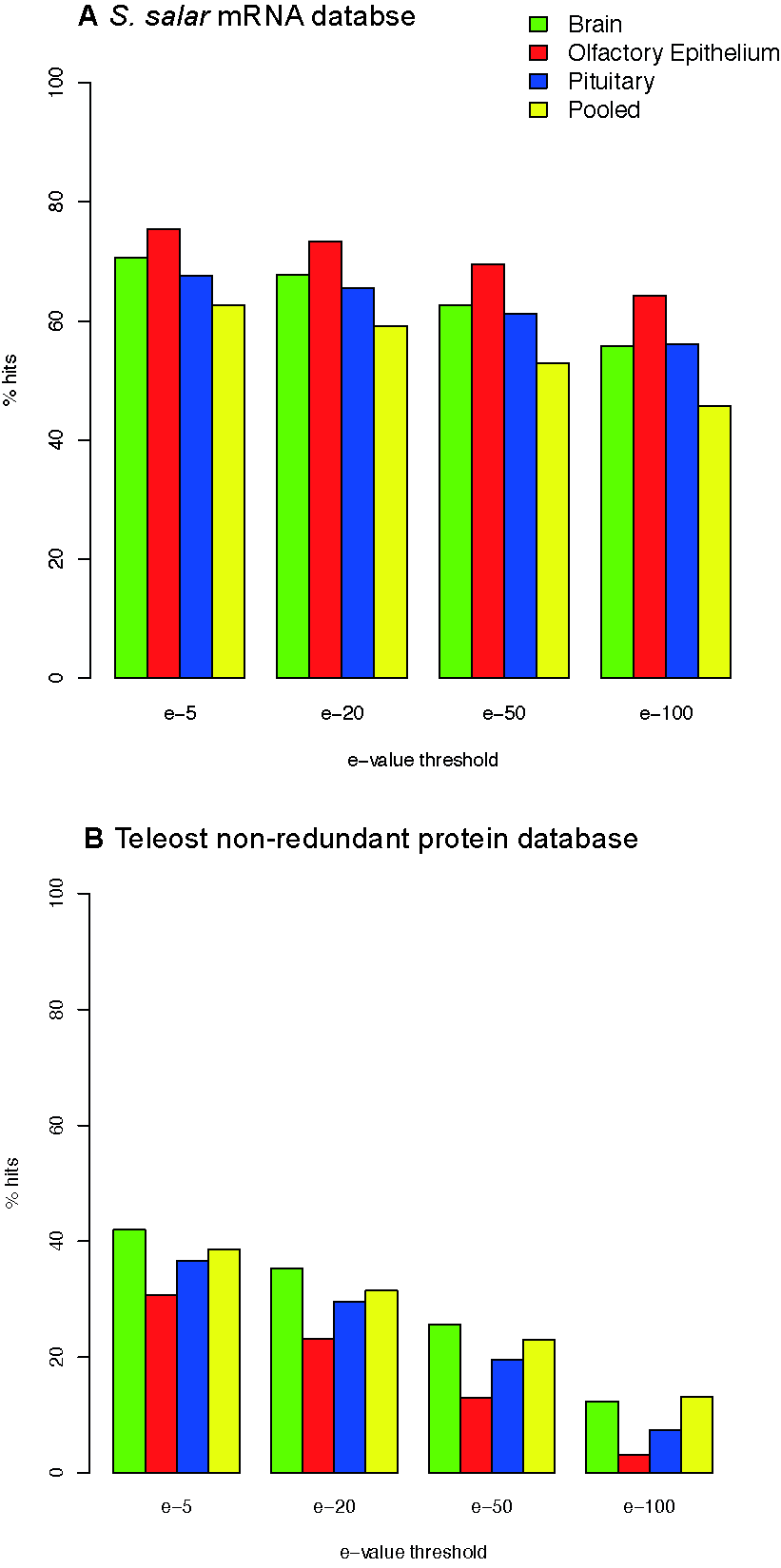


*Figure S4.1*: Congruence of genes expressed in each transcriptome assembly with existing genomic resources (additional to Fig. 3 in the main manuscript): **A)** *S. salar* mRNA database from the NCBI; **B)** Teleost non-redundant protein database (Benson et al. 2006). The percentage of isogroups with significant BLAST hits are given on the y-axis, and the E-value threshold is given on the x-axis

**Supplementary Section 5: Paralog searches at different threshold values**

This section contains the runs of the analysis carried out in Results Section 2.2. Table S5.1 summarised the output. Figures S5.1 to S5.8 are re-drawings of Fig. 5 from the manuscript at the different parameter levels. Fig S5.9 gives a summary of the Ka/Ks values at the different threshold levels.

*Table S5.1: Paralog pair summaries:*

| **Thresholds (%ID/bp)** | **70/300** | **70/600** | **75/300** | **75/600** | **80/300** | **80/600** | **85/300** | **85/600** |
| --- | --- | --- | --- | --- | --- | --- | --- | --- |
| *Total pairs found* | 2947 | 1399 | 2930 | 1387 | 2451 | 1147 | 1386 | 648 |
| *Pairs on different contig* | 2888 | 1381 | 2871 | 1369 | 2393 | 1129 | 1338 | 634 |
| *Pairs on same contig* | 59 | 18 | 59 | 18 | 58 | 18 | 48 | 14 |
| *Overlapping hits* | 58 | 17 | 58 | 17 | 57 | 17 | 48 | 14 |
| *Non-overlapping hits* | 1 | 1 | 1 | 1 | 1 | 1 | 0 | 0 |
| *Total paralogs* | 2889 | 1382 | 2872 | 1370 | 2394 | 1130 | 1338 | 634 |
| *Mean %ID* | 84.74 | 84.60 | 84.80 | 84.70 | 86.11 | 86.07 | 88.89 | 88.81 |
| *Median %ID* | 84.57 | 84.535 | 84.62 | 84.595 | 85.57 | 85.585 | 88.44 | 88.395 |
| *Mean Ks* | 0.39 | 0.37 | 0.39 | 0.36 | 0.35 | 0.34 | 0.28 | 0.25 |
| *Median Ks* | 0.25 | 0.25 | 0.25 | 0.25 | 0.24 | 0.24 | 0.23 | 0.22 |
| *Mean Ka/Ks* | 0.38 | 0.31 | 0.38 | 0.31 | 0.38 | 0.29 | 0.32 | 0.25 |
| *Median Ka/Ks* | 0.22 | 0.17 | 0.22 | 0.17 | 0.21 | 0.16 | 0.18 | 0.12 |
| *WGD paralogs* | 95 | 56 | 95 | 56 | 79 | 45 | 46 | 27 |
| *WGD Mean %ID* | 84.94 | 84.60 | 84.94 | 84.60 | 86.33 | 86.17 | 88.96 | 88.56 |
| *WGD Median %ID* | 84.90 | 84.94 | 84.90 | 84.94 | 86.28 | 86.39 | 88.90 | 88.66 |
| *WGD Mean Ks* | 0.53 | 0.54 | 0.53 | 0.54 | 0.40 | 0.39 | 0.29 | 0.21 |
| *WGD Median Ks* | 0.28 | 0.27 | 0.28 | 0.27 | 0.27 | 0.27 | 0.23 | 0.21 |
| *WGD Mean Ka/Ks* | 0.21 | 0.14 | 0.21 | 0.14 | 0.21 | 0.15 | 0.17 | 0.13 |
| *WGD Median Ka/Ks* | 0.11 | 0.09 | 0.11 | 0.09 | 0.12 | 0.10 | 0.11 | 0.10 |
| *LGD paralogs* | 58 | 37 | 58 | 37 | 57 | 36 | 43 | 27 |
| *LGD Mean %ID* | 87.59 | 87.45 | 87.59 | 87.45 | 87.74 | 87.69 | 89.24 | 89.10 |
| *LGD Median %ID* | 87.65 | 87.44 | 87.65 | 87.44 | 87.86 | 87.68 | 88.92 | 88.91 |
| *LGD Mean Ks* | 0.23 | 0.23 | 0.23 | 0.23 | 0.23 | 0.22 | 0.23 | 0.21 |
| *LGD Median Ks* | 0.22 | 0.21 | 0.22 | 0.21 | 0.21 | 0.21 | 0.20 | 0.20 |
| *LGD Mean Ka/Ks* | 0.37 | 0.33 | 0.37 | 0.33 | 0.35 | 0.30 | 0.30 | 0.21 |
| *LGD Median Ka/Ks* | 0.25 | 0.21 | 0.25 | 0.21 | 0.24 | 0.16 | 0.19 | 0.13 |
| *Shapiro wilks for %ID* | <0.0001 | <0.0001 | <0.0001 | <0.0001 | <0.0001 | <0.0001 | <0.0001 | <0.0001 |
| *%ID Wilcox: W* | 1861.50 | 683.00 | 1861.50 | 683.00 | 1779.00 | 636.00 | 945.00 | 337.00 |
| *%ID Wilcox: p-value* | 0.00078 | 0.00566 | 0.00078 | 0.00566 | 0.03737 | 0.09933 | 0.72098 | 0.64304 |
| *Shapiro Wilks for Ks* | <0.0001 | <0.0001 | <0.0001 | <0.0001 | <0.0001 | <0.0001 | <0.0001 | <0.0001 |
| *Ks_t-test: t* | 2.92 | 2.07 | 2.92 | 2.07 | 2.46 | 1.83 | 1.38 | 0.55 |
| *Ks t-test: df* | 49.12 | 39.51 | 49.12 | 39.51 | 41.42 | 34.07 | 29.86 | 19.22 |
| *Ks t-test: p-value* | 0.00521 | 0.04530 | 0.00521 | 0.04530 | 0.01811 | 0.0762 | 0.1766 | 0.59175 |
| *Ks Wilcox: W* | 582.00 | 271.00 | 582.00 | 271.00 | 498.00 | 244.00 | 242.00 | 107.00 |
| *Ks Wilcox:_p-value* | 0.01686 | 0.16774 | 0.01686 | 0.16774 | 0.03753 | 0.19136 | 0.36876 | 0.94862 |

*Figure S5.1: Thresholds ID = 70%, length = 300bp*

*Figure S5.2: Thresholds ID = 70%, length = 600bp*

*Figure S5.3: Thresholds ID = 75%, length = 300bp*

*Figure S5.4: Thresholds ID = 75%, length = 600bp*

*Figure S5.5: Thresholds ID = 80%, length = 300bp*

*Figure S5.6: Thresholds ID = 80%, length = 600bp*

*Figure S5.7: Thresholds ID = 85%, length = 300bp*

*****Figure S5.8: Thresholds ID = 85%, length = 600bp*

*Figure S5.9: Ka/Ks values at all thresholds with all data included*

**Supplementary Section 6: Analysis of functional changes in paralog pairs when compared to *S. salar* unigene database and a salmonid mRNA database.**


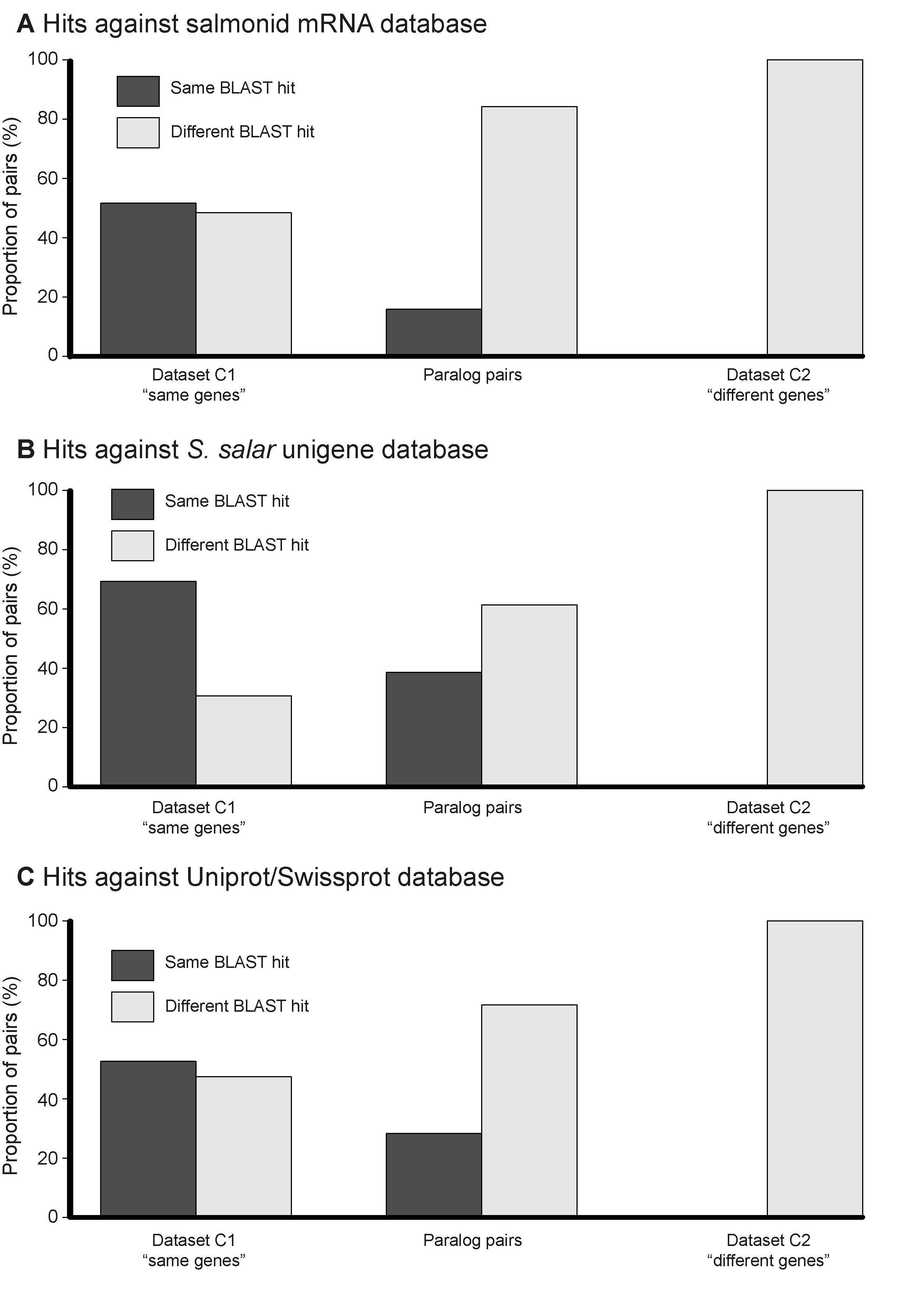


*Figure S6.1*: Analysis of functional changes in putative paralog pairs. **A)** BLASTn hits for the two control datasets (C1: “same genes” and C2: “different genes”) and the real dataset of paralog pairs (identified in section 2 in main text), When compared against an (**A**) Genbank salmonid mRNA database, (**B**) an NCBI Unigene *S. salar* database, and (**C**) a Uniprot/Swissprot database. In all cases, the differences between the data sets is significant (vs. Salmonid mRNA: *χ^2^* = 2292.9, d.f. = 2, *p* < 0.0001; vs. *S. salar* unigene: *χ^2^* = 2765.4, d.f. = 2, *p* < 0.0001; vs UniProt/SwissProt: *χ^2^* = 1944.1, d.f. = 2, *p* < 0.0001). Complementary to Fig. 7 in the main manuscript.

**
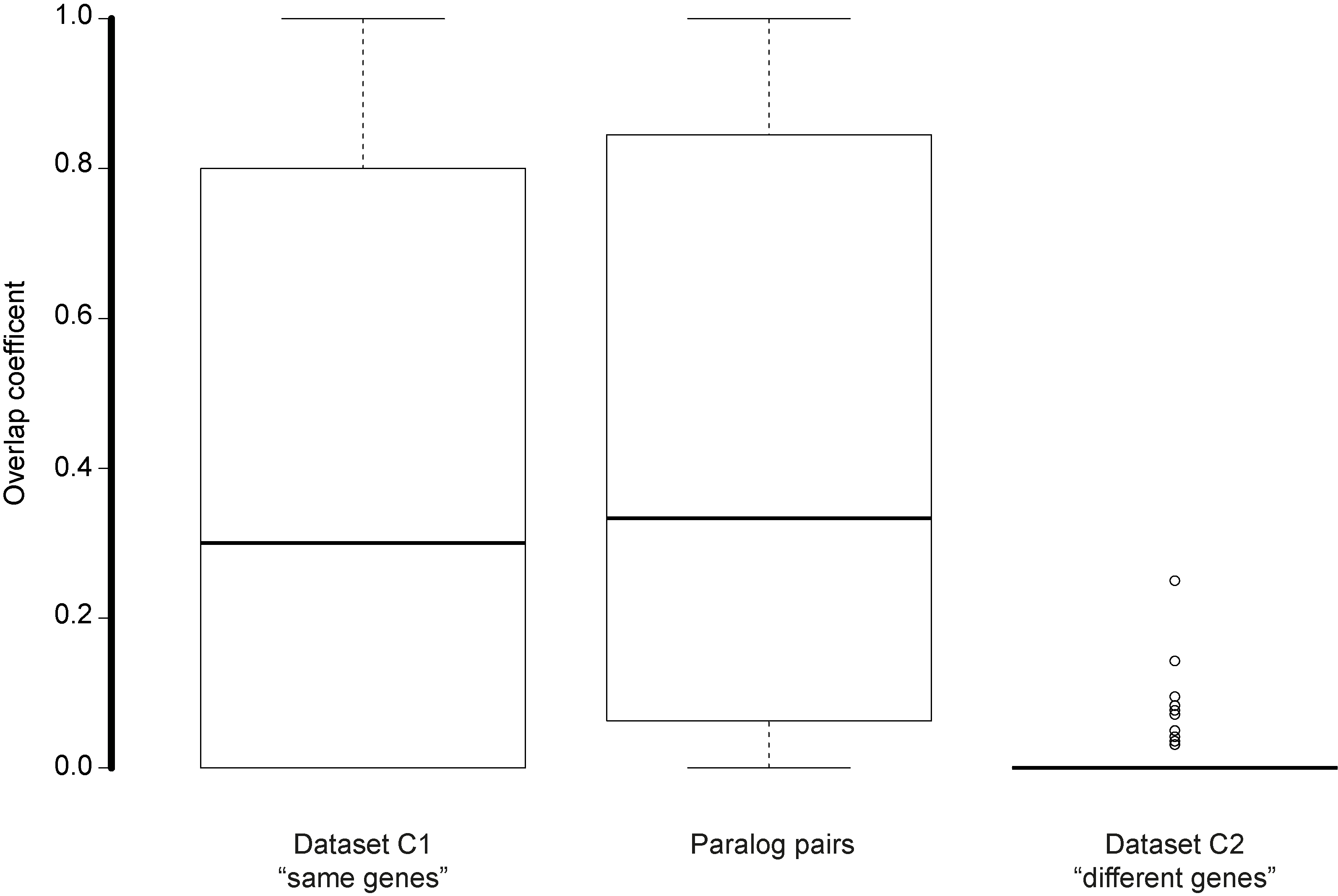
**

*Figure S6.2*: Overlap of GO terms retrieved from Uniprot hits between pairs of sequences. Sequence pairs where the same UniProt BLAST hit was obtained were removed from the analysis. This analysis included 109 pairs from the Dataset C1, 143 pairs from the duplicate pairs, and 139 pairs from Dataset C2.

**Supplementary Section 7: Changes in function for local gene duplication- and whole genome derived duplications**

**
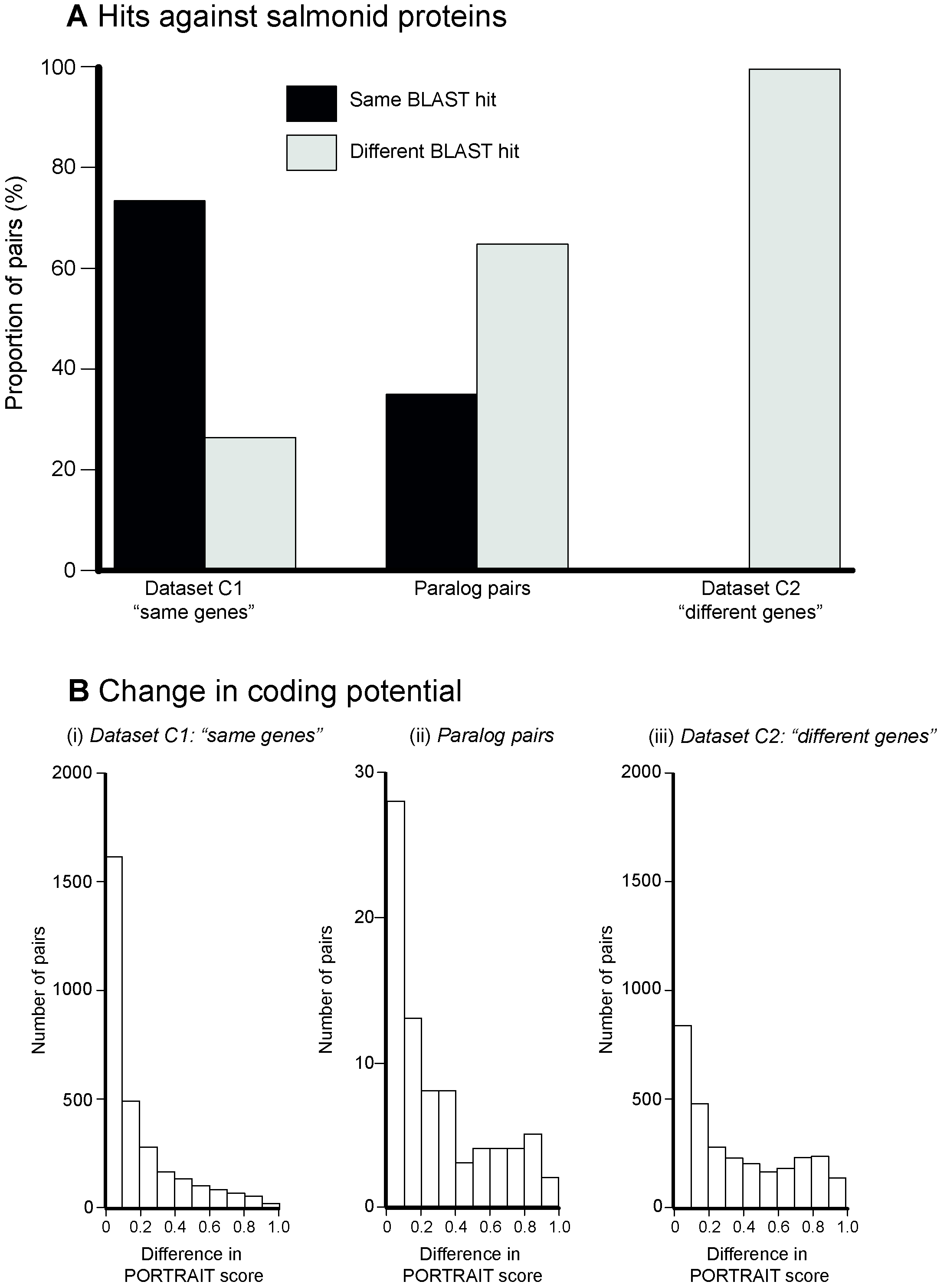
**

*Figure S7.1*: Analysis of functional changes in paralog pairs from whole genome duplication event. **A)** BLAST hits for the two control datasets (C1: “same genes” and C2: “different genes”) and the real dataset of paralog pairs (identified in section 2), when compared against an NCBI salmonid protein database. The proportion of pairs is given on the y-axis. In the majority of sequence pairs, neither sequence had a positive BLASTx hit and are not shown here. Differences were significant between the groups: *χ^2^* = 1588.5, *df* = 4, *p* < 0.0001. **B)** The absolute difference in coding potential for each sequence pair across the three datasets (represented by absolute difference within paralog pairs in their respective PORTRAIT scores (Arrial et al. 2009)). Again all differences were significant Mann-Whitney: vs C1: *W* = 2717591, *p* < 0.0001; vs C2: *W* = 133307, *p* = 0.035.

**
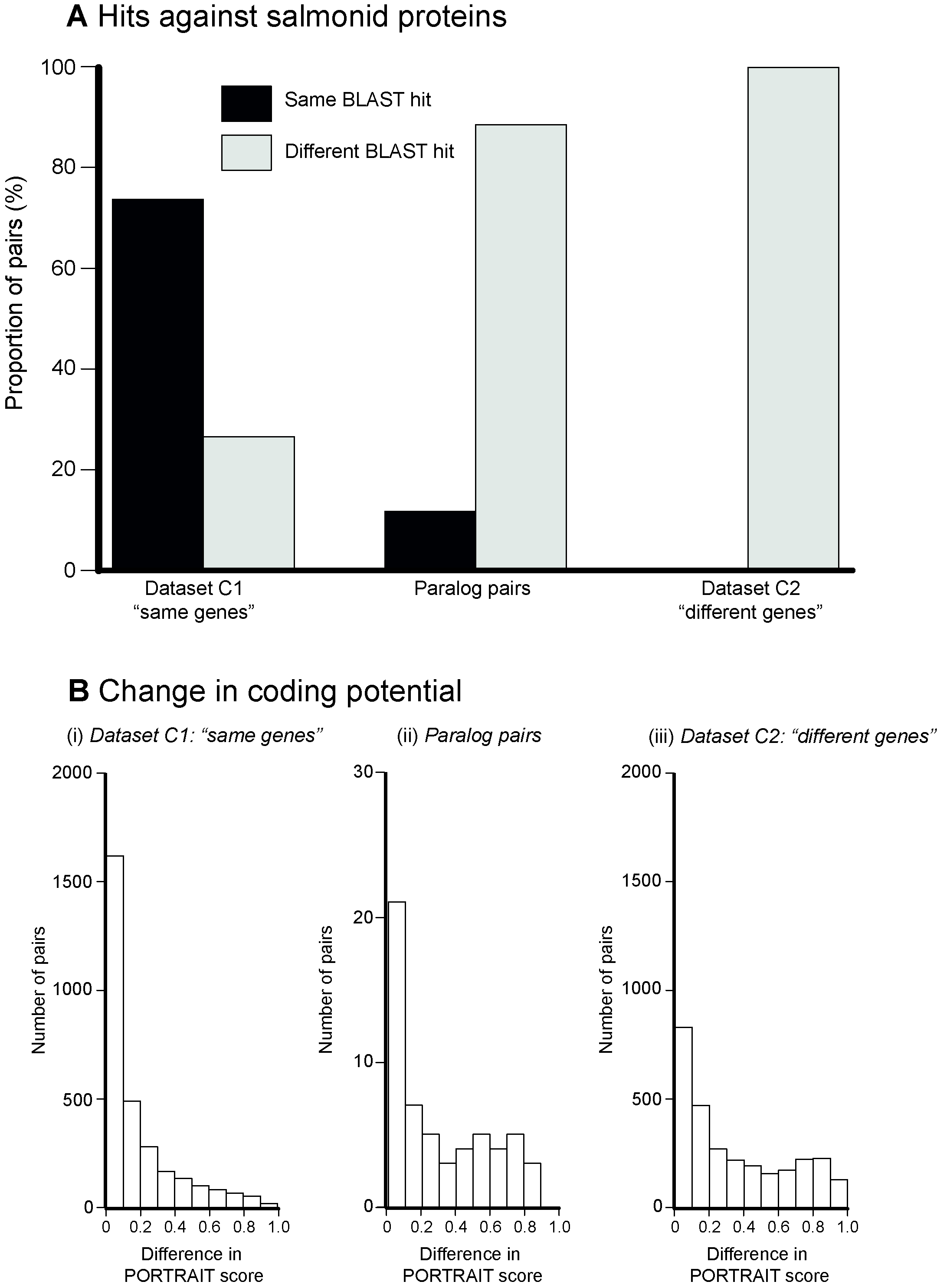
**

*Figure S7.2*: Analysis of functional changes in paralog pairs from local duplication events. **A)** BLAST hits for the two control datasets (C1: “same genes” and C2: “different genes”) and the real dataset of paralog pairs (identified in section 2), when compared against an NCBI salmonid protein database. The proportion of pairs is given on the y-axis. In the majority of sequence pairs, neither sequence had a positive BLASTx hit and are not shown here. Differences were significant between the groups: *χ^2^* = 1608.1, *df* = 4, *p* < 0.0001. **B)** The absolute difference in coding potential for each sequence pair across the three datasets (represented by absolute difference within paralog pairs in their respective PORTRAIT scores) (Arrial et al. 2009). Again differences were significant against database C1 (Mann-Whitney *W* = 2717591, *p* < 0.0001), but not against database C2 (*W* = 92865, *p* = 0.20).

*
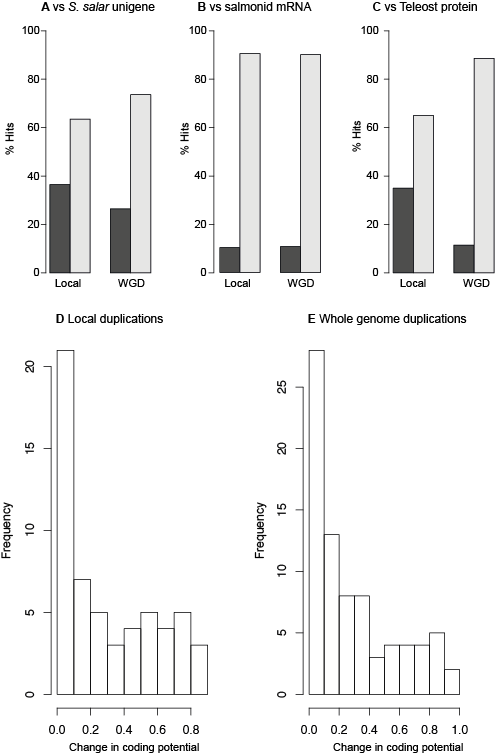
*

*Figure S7.3:* Changes in function between local duplication events and whole genome duplication (WGD) events. No significant difference in proportion of same BLAST hits are seen between local duplication and WGD events when compared against: **A:** *S. salar* NCBI unigene data base (*χ^2^* = 1.0092,  *p* = 0.32); **B**: A salmonid mRNA database downloaded from genbank (*χ^2^* = 0.006 ,  *p* = 0.94); **C**: Teleost NCBI protein (*χ^2^* = 4.47,  *p* = 0.035). No observable differences were seen in changes in coding potential within pairs between (**D**) local duplication derived paralogs and between (**E**) WGD even derived paralogs (permutations tests for median and inter-quartile range: *p* > 0.5; Mann-Whitney: *W* = 2174, *p* = 0. 73).

References

Anon. 2011. 454 Sequencing System Software Manual Version 2.6. Mannheim, Germany: Roche Diagnostics GmbH, Roche Applied Science

Arrial RT, Togawa RC, Brigido MDM. 2009. Screening non-coding RNAs in transcriptomes from neglected species using PORTRAIT: case study of the pathogenic fungus Paracoccidioides brasiliensis. BMC Bioinformatics 10:239.

Benson DA, Karsch-Mizrachi I, Lipman DJ, Ostell J, Wheeler DL. 2006. GenBank. Nucleic Acids Res. 34:D16–20.

Camon E, Magrane M, Barrell D, et al. 2004. The Gene Ontology Annotation (GOA) Database: sharing knowledge in Uniprot with Gene Ontology. Nucleic Acids Res. 32:D262–6.

Choi J-H, Kijimoto T, Snell-Rood E, Tae H, Yang Y, Moczek AP, Andrews J. 2010. Gene discovery in the horned beetle Onthophagus taurus. BMC Genomics 11:703.

Conesa A, Götz S, García-Gómez JM, Terol J, Talón M, Robles M. 2005. Blast2GO: a universal tool for annotation, visualization and analysis in functional genomics research. Bioinformatics 21:3674–3676.

Davidson WS, Koop BF, Jones SJM, Iturra P, Vidal R, Maass A, Jonassen I, Lien S, Omholt SW. 2010. Sequencing the genome of the Atlantic salmon (Salmo salar). Genome Biol. 11:403.

Grabherr MG, Haas BJ, Yassour M, et al. 2011. Full-length transcriptome assembly from RNA-Seq data without a reference genome. Nat. Biotechnol. 29:644–652.

Leong JS, Jantzen SG, Schalburg KR Von, et al. 2010. Salmo salar and Esox lucius full-length cDNA sequences reveal changes in evolutionary pressures on a post-tetraploidization genome. BMC Genomics 11:279.

The UniProt Consortium. 2012. Reorganizing the protein space at the Universal Protein Resource (UniProt). Nucleic Acids Res. 40:D71–D75.
